# Capsid-Specific Antibody Responses of Domestic Pigs Immunized with Low-Virulent African Swine Fever Virus

**DOI:** 10.3390/vaccines11101577

**Published:** 2023-10-10

**Authors:** Priscilla Y. L. Tng, Laila Al-Adwani, Egle Pauletto, Joshua Y. K. Hui, Christopher L. Netherton

**Affiliations:** 1 The Pirbright Institute, Ash Road, Pirbright, Woking GU24 0NF, UK; laila.al-adwani@pirbright.ac.uk (L.A.-A.); e.pauletto.19@abdn.ac.uk (E.P.); joshua.hui@pirbright.ac.uk (J.Y.K.H.); 2 The Institute of Medical Sciences, Foresterhill, University of Aberdeen, Aberdeen AB25 2ZD, UK

**Keywords:** ASFV, humoral responses, ASFV capsid proteins, ASFV vaccines, ASFV immunity, antigen discovery, luciferase antibody capture assay, luciferase immunoprecipitation assay, viral hemorrhagic fever

## Abstract

African swine fever (ASF) is a lethal disease in pigs that has grave socio-economic implications worldwide. For the development of vaccines against the African swine fever virus (ASFV), immunogenic antigens that generate protective immune responses need to be identified. There are over 150 viral proteins—many of which are uncharacterized—and humoral immunity to ASFV has not been closely examined. To profile antigen-specific antibody responses, we developed luciferase-linked antibody capture assays (LACAs) for a panel of ASFV capsid proteins and screened sera from inbred and outbred animals that were previously immunized with low-virulent ASFV before challenge with virulent ASFV. Antibodies to B646L/p72, D117L/p17, M1249L, and E120R/p14.5 were detected in this study; however, we were unable to detect B438L-specific antibodies. Anti-B646L/p72 and B602L antibodies were associated with recovery from disease after challenges with genotype I OUR T88/1 but not genotype II Georgia 2007/1. Antibody responses against M1249L and E120R/p14.5 were observed in animals with reduced clinical signs and viremia. Here, we present LACAs as a tool for the targeted profiling of antigen-specific antibody responses to inform vaccine development.

## 1. Introduction

The on-going African swine fever (ASF) panzootic in domestic pigs and wild boar is caused by the ASF virus (ASFV) [1,2]. It is a contagious and lethal hemorrhagic disease that is of international concern. Due to the absence of approved and effective vaccines or treatments, the main control measures involve slaughter and movement control of swine [3], resulting in high economic losses and impacting global food security. ASFV is a large, double-stranded virus that has a 170 to 193 kb genome encoding over 150 genes [4,5], many of which are uncharacterized [1,5].

Protective immune responses after recovery from ASFV infection are poorly understood. Vaccine development efforts are mostly focused on the development of live attenuated viruses (LAVs) [6,7,8,9,10]. Although these afford good protection, they are not DIVA (differentiating infected from vaccinated animals)-compliant and there are potential safety concerns [3,9]. Unlike LAVs, subunit vaccines only encode for selected viral antigens and have an inherently safe design that is DIVA-compliant. However, the combinations that have been developed and tested so far offer varied protection [11,12,13,14]. Generating good T-cell responses has generally been the focus of ASFV vaccine development efforts [11,13,15] although both cellular and humoral immune responses are important for robust protection against ASFV. Antigen-specific cellular immune responses in animals immunized with low-virulent ASFV have previously been described [11]. Despite studies demonstrating the importance of anti-ASFV antibodies in disease protection [16,17,18], antigen-specific antibody responses to ASFV have remained largely uncharacterized due to the difficulties in detecting neutralizing antibodies [19,20] and the lack of tools.

ASFV-specific antibody responses are typically measured with fixed virus-infected cells or lysates that only provide a broad overview of the antibody responses. Commercial ASFV antigen-specific ELISAs are limited to a small number of ASFV antigens, like CP204L/p30 and B646L/p72. Furthermore, the development of ASFV antigen-specific ELISAs has mostly focused on diagnostic purposes with highly immunogenic antigens [21,22,23,24,25,26]. Recombinant protein production and purification is a core prerequisite for ELISA development, and due to the structure and immunomodulatory nature of many ASFV proteins [27], high yields in mammalian expression systems with proper post-translational modifications can prove difficult to achieve. Hence, there is a need to explore alternative antibody detection assays that can facilitate antigen-specific antibody screening for antigen discovery in subunit vaccine developments.

Luciferase-based antibody diagnostics have previously been reported for porcine diseases using luciferase immunoprecipitation systems (LIPS) [28,29] and luciferase-linked antibody capture assays (LACAs) [30]. Both LIPS and LACAs detect and quantify antigen-specific antibodies indirectly through the capture of antibodies that are bound to recombinant luciferase-tagged proteins of interest [30,31]. The capture of antibodies is typically achieved with protein A. Furthermore, unlike ELISAs, these assays do not require protein purification and allow the use of crude cell lysates [31]. Compared to LIPS, LACAs are a more cost-effective approach for screening a large number of samples with multiple antigens [30], especially for the purposes of antigen discovery.

ASFV is a highly complex virus with many structural proteins involved in the assembly of its multi-layered structure. Recently, the ASFV capsid structure has been resolved by three separate groups, highlighting the proteins involved in capsid construction [32,33,34]. B646L/p72 is the major capsid protein and the most abundant protein within the capsid [35]. It is highly immunogenic and conserved, hence its use in routine serological diagnostics [36] and genotyping [37]. To obtain B646L/p72 proteins that have a native conformation, co-expression of the virally encoded B602L chaperone is required [38,39]. B602L is not present in virus replication sites, and antibodies against B602L can be detected in recovered pigs [40,41]. D117L/p17, a minor capsid protein that is a component of the internal envelope [33], is also essential for virus morphogenesis [42]. D117L/p17 is immunogenic [43] and may have immunomodulatory abilities [44]. B438L/p49 is required for the assembly of icosahedral virions and is located at the vertices of the capsid [34,45]. Previous work has associated B438L/p49 with low immunogenicity [46]. M1249L is a large structural protein that may be involved in building the framework of the capsid [32,33]. E120R/p14.5 is a minor capsid protein that is associated with B646L/p72 and is essential for virus dissemination [47]. It has been suggested that E120R/p14.5 may have DNA-binding properties [48]. Similar to other capsid proteins, host immunomodulatory functions have been associated with E120R [49]. To our knowledge, porcine antibody responses to M1249L and E120R have not been described before.

In this study, we sought to profile the dynamics of antigen-specific antibodies targeting capsid proteins using LACAs for a panel of ASFV capsid and associated proteins: (1) B646L/p72 (with and without co-expression of B602L); (2) D117L/p17; (3) B438L/p49; (4) M1249L; (5) E120R/p14.5; and (6) the chaperone B602L since the ASFV capsid structure has been resolved by multiple groups. We characterized the longitudinal humoral responses using samples from our previous study [50] where we immunized inbred Babraham pigs and outbred domestic pigs with the low-virulent OURT88/3 (genotype I) isolate. We produced a CP204L/p30 LACA to confirm the performance of LACAs for measurement of antibody responses as a comparison to the commercial CP204L/p30 competitive ELISA used in our previous work [50]. We were able to identify antigen-specific antibodies against most of the antigens tested and distinguish responses associated with recovery from virulent ASFV challenges.

## 2. Materials and Methods

### 2.1. Sera Samples from Animal Experiments and Ethics Statement

**Figure 1:**
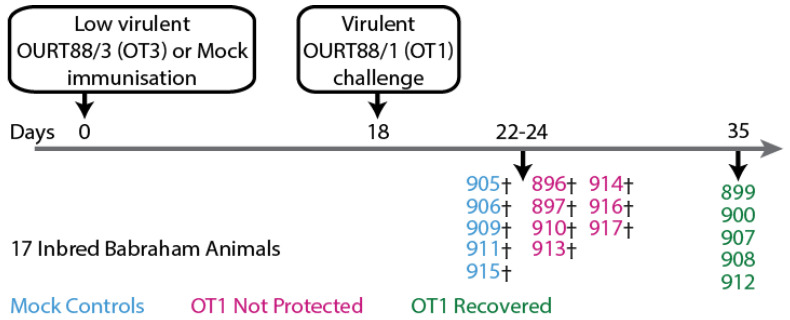
Schematic representation of experiment involving large, white inbred Babraham pigs that were immunized with low-virulent OURT88/3 (OT3) before challenge with virulent OURT88/1 (OT1), as published previously [50]. Partial protection from challenge with related ASFV was observed. (†) indicates that the animal was euthanized before the conclusion of the experiment because it had reached its humane endpoint.

**Figure 2:**
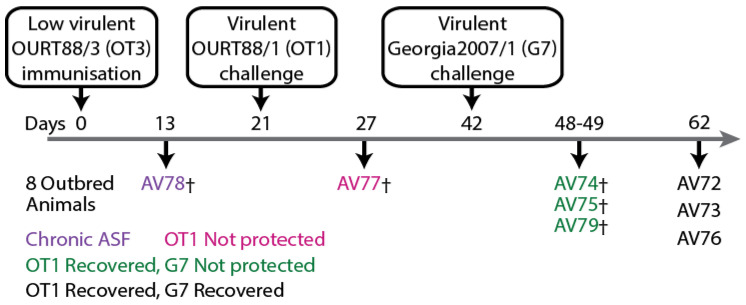
Schematic representation of experiment involving outbred domestic pigs that were immunized with low-virulent OURT88/3 and challenged with the related virulent OURT88/1. Survivors were then challenged further with the heterologous Georgia 2007/1 [50]. Partial protection from challenge with homologous and heterologous virulent ASFV was observed. (†) indicates that the animal was euthanized before the conclusion of the experiment because it had reached its humane endpoint.

Sera samples were obtained from two experiments that were published previously [50]. Both animal experiments were performed under the jurisdiction of the Home Office Animals (Scientific Procedures) Act from 1986; approval was obtained from the Animal Welfare and Ethical Review Board (AWERB) of the Pirbright Institute. All procedures were conducted by Personal License holders competently trained under the Project License PPL70/8852. The first experiment (Figure 1) consisted of twelve inbred Babraham pigs (animal tag numbers 896, 897, 899, 900, 907, 908, 910, 912, 913, 914, 916, and 917) experimentally immunized with low-virulent ASFV strain OURT88/3 and five mock control animals (animal tag numbers 905, 906, 909, 911, and 915) immunized with PBS before challenge with the homologous virulent ASFV strain OURT88/1. In the second experiment (Figure 2), eight outbred domestic pigs (animal numbers AV72—AV79) were experimentally immunized with OURT88/3 before challenge with virulent OURT88/1. Surviving animals were thereafter challenged with the heterologous ASFV strain Georgia 2007/1.

### 2.2. Plasmids

Open reading frames for the following proteins: B646L/p72, B602L, D117L/p17, M1249L, and E120R/p14.5, were synthesized based on the sequences from ASFV Georgia2007/1 (GenBank Accession No. NC_044959). Open reading frames (Supplementary Table S1) were pig codon optimized and cloned into a modified pNeoSec vector [51] (kindly provided by Prof. Raymond Owens, Protein Production UK (PPUK)) without the RPTP secretion signal. Proteins were expressed under the control of the synthetic CAG promoter, and all proteins were tagged on the C-terminal end with nanoluciferase (Nluc, Supplementary Table S2) derived from the deep-sea shrimp *Oplophorus gracilirostris*. Nluc is a smaller luciferin that is able to metabolize coelenterazine—similar to *Renilla* luciferase—but with higher levels of luminescence [52]. The smaller size of Nluc (approximately 19.1 kDa), compared to *Renilla* luciferase (36 kDa), reduces the possibility of steric hindrance of potential epitopes. Plasmids were amplified in DH5α derivative-competent cells (New England Biolabs, Ipswich, MA, USA) and isolated for transfection with Miraprep as described by Pronobis et al. [53].

### 2.3. Cell Culture and Transfections

Since ASFV replicates in porcine cells, we expressed Nluc-tagged recombinant ASFV proteins in mammalian cell lines to ensure that mammalian post-translational modifications were applied appropriately. HEK293T cells were used for protein production. Cells were maintained at 37 °C with 5% CO_2_ and humidity control in Dulbecco’s modified Eagle’s medium (Gibco-Thermo Fisher Scientific, Waltham, MA, USA) supplemented with 10% fetal bovine serum (FCS, Life Science Production, Sandy, Bedfordshire, UK) and 100 I.U./mL of penicillin with 100 µg/mL of streptomycin (Gibco-Thermo Fisher Scientific, Waltham, MA, USA).

HEK293T cells were seeded into T175 flasks at a density of 4 × 10^6^ cells and transfected when they reached approximately 70% confluence. All plasmids were transfected using TransIT-LT1 (Mirius Bio LLC, Madison, WI, USA) according to manufacturer’s instructions. Cells were maintained for 72 h after transfection before harvesting. Cells were pelleted and washed once with 1× phosphate-buffered saline (PBS) before lysis with 1× *Renilla* Luciferase Assay (RLA) lysis buffer (Promega, Madison, WI, USA) with 1× cOmplete protease inhibitor cocktail (Roche, Basal, Switzerland). Lysates were incubated for 15 min with agitation before one freeze–thaw cycle to ensure complete lysis of cells. After clarification at 13,200× *g* (4 °C), lysates were measured with RLA (Promega, Madison, WI, USA) to determine Nluc activity according to manufacturer’s instructions. Lysates were then aliquoted and stored below −70 °C until use in assays. The same lysates were used for all samples within an experiment.

### 2.4. LACA

**Figure 3:**
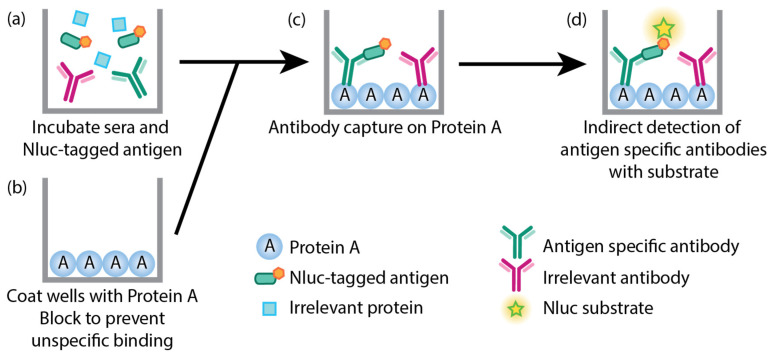
Schematic of modified LACA developed to study ASFV antigen-specific antibody responses of immunized and challenged animals. (**a**) Sera samples are incubated with cell lysates containing a mixture of specific Nluc-tagged ASFV proteins of interest and irrelevant proteins. (**b**) Plates are coated with protein A and blocked to prevent unspecific binding. (**c**) Nluc-tagged antigens bound to their respective antibodies are then captured on protein A, and (**d**) the presence of antigen-binding antibodies is indirectly measured through the Nluc activity. Irrelevant antibodies bound to protein A are not detected since these do not bind the Nluc-tagged antigens.

A modified version of LACA [30] was used in this study (Figure 3). Briefly, 96-well LumiNunc opaque white plates (Thermo Fisher Scientific, Waltham, MA, USA) were coated with 8 µg/mL of protein A from *Staphylococcus aureus* (Merck, Darmstadt, Germany) overnight in 100 µL carbonate/bicarbonate buffer (Merck, Darmstadt, Germany) at 4 °C. Plates were then washed three times with 0.5% (*v*/*v*) Triton-X100 (Merck, Darmstadt, Germany) in 1× PBS (wash buffer). Thereafter, plates were blocked with 5% skim milk in wash buffer (block buffer) for two hours. Antigen lysates were diluted to 2 × 10^7^ ALU/mL in block buffer and sera samples were diluted 1:50 in block buffer before incubation with diluted antigen lysates at a ratio of 1:1 (sera samples have a final dilution of 1:100 in a final volume of 100 µL) for one hour with agitation. Block buffer was then removed from the white plates, and the sera–antigen lysate mixes were transferred into the blocked plates and incubated for one hour with agitation. Plates were washed six times with wash buffer and twice with 1×PBS before measurement with RLA using a Cytation 3 multi-mode reader (BioTek Instruments, Winooski, VT, USA). All incubations were performed at room temperature unless indicated. FCS was used as a negative control on each plate, and data were presented as a ratio of luciferase activity of each sample to the luciferase activity of the negative control (P/N ratio). The cutoff value for the P/N ratio of each protein was calculated from the mean and 3× standard deviation of all negative sera samples in each experiment.

### 2.5. Statistics

Statistical analyses were performed with GraphPad Prism 9 (GraphPad Software, Boston, MA, USA) on the responses of inbred Babraham animals. Statistical differences between Babraham pigs immunized with PBS (n = 5) immunized Babraham animals that were protected (n = 5) and not protected (n = 7) after virulent challenge were tested using the mixed-effects model (REML) with Tukey’s multiple comparison test. Time points where there were at least four data points for each group were used for this analysis. Data were transformed to fit a normal distribution as appropriate before models were run, and diagnostic plots of residuals were checked to ensure that model assumptions were met. A *p*-value of less than 0.05 was considered statistically significant. Graphs were plotted with GraphPad Prism 9 and arranged with Illustrator CS6 (Adobe, San Jose, CA, USA).

## 3. Results

We previously reported on the humoral and cellular responses of inbred Babraham animals and domestic pigs that were immunized with low-virulent OURT88/3 before challenge with virulent ASFV [11,50]. Using whole ASFV fixed-cell and commercial ELISAs, we found that the general humoral responses of inbred Babraham animals were weakly associated with recovery after challenge with virulent ASFV OURT88/1 [50]. We did not, however, identify antigens that were associated with protection due to the lack of available tools. To probe the antigen-specific antibody responses of samples collected from our previous study, we developed a modified version of LACAs [30] (Figure 3). Recombinant ASFV capsid protein expression was confirmed with confocal imaging (Supplementary Figures S1 and S2) and Western blot (Supplementary Figure S3). Two assays were developed for the major capsid protein B646L/p72: one where B646L/p72 is co-expressed with an untagged chaperone B602L, which has been described as essential for the proper conformation of B646L/p72 [35,38,39], and one where B646L/p72 is expressed alone. The co-transfection ratio of B646L/p72 and B602L to develop an assay using recombinant B646L/p72 with a conformation that is reminiscent of its native conformation was determined empirically with confocal imaging; binding of the conformation-dependent 4H3 antibody [54] was only observed at a ratio of 1:1 (B646L/p72: B602L, Supplementary Figure S4). In this work, we refer to antibodies that target B646L/p72 co-expressed with B602L as B646L/p72-B602L antibodies and antibodies that bind to non-conformational B646L/p72 (no co-expression of B602L) targets as B646L/p72 antibodies.

### 3.1. Antigen-Specific Antibody Responses of Inbred Babraham Pigs

**Figure 4:**
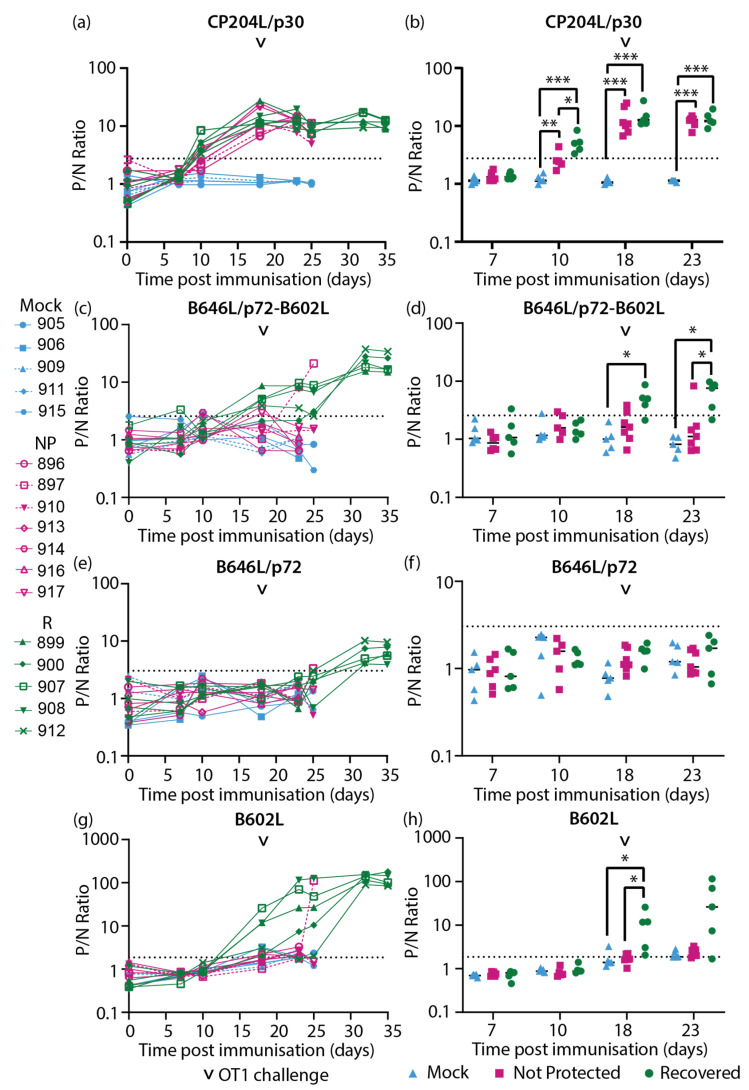
Longitudinal antibody responses of inbred Babraham animals to ASFV recombinant proteins (**a**,**b**) CP204L/p30, (**c**,**d**) B646L/p72 co-expressed with chaperone B602L, (**e**,**f**) B646L/p72, and (**g**,**h**) B602L chaperone detected with antigen-specific LACAs at selected days post-immunization (dpi). (**a**,**c**,**e**,**g**) Antigen-specific antibody kinetics of each animal are plotted. (**b**,**d**,**f**,**h**) Antibody responses consolidated as a group at each relevant time point are plotted. Only time points where there were four data sets or more were included in this analysis. Lines indicate the mean. Each data point corresponds to a single animal. The point of challenge with virulent OURT88/1 (OT1, 18 dpi) is denoted by the arrowhead in each graph. P/N Ratio: ratio of luciferase activity of each sample to the luciferase activity of the negative control. Dashed line indicates the cutoff determined from the mean and 3x standard deviation of all negative sera samples in each experiment. Blue, Mock: mock control animals immunized with PBS, Pink, NP: OURT88/3 immunized animals that were not protected from OURT88/1, Green, R: OURT88/3 immunized animals that recovered from OURT88/1. * *p* < 0.05, ** *p* < 0.01, *** *p* < 0.005, mixed effects model.

**Figure 5:**
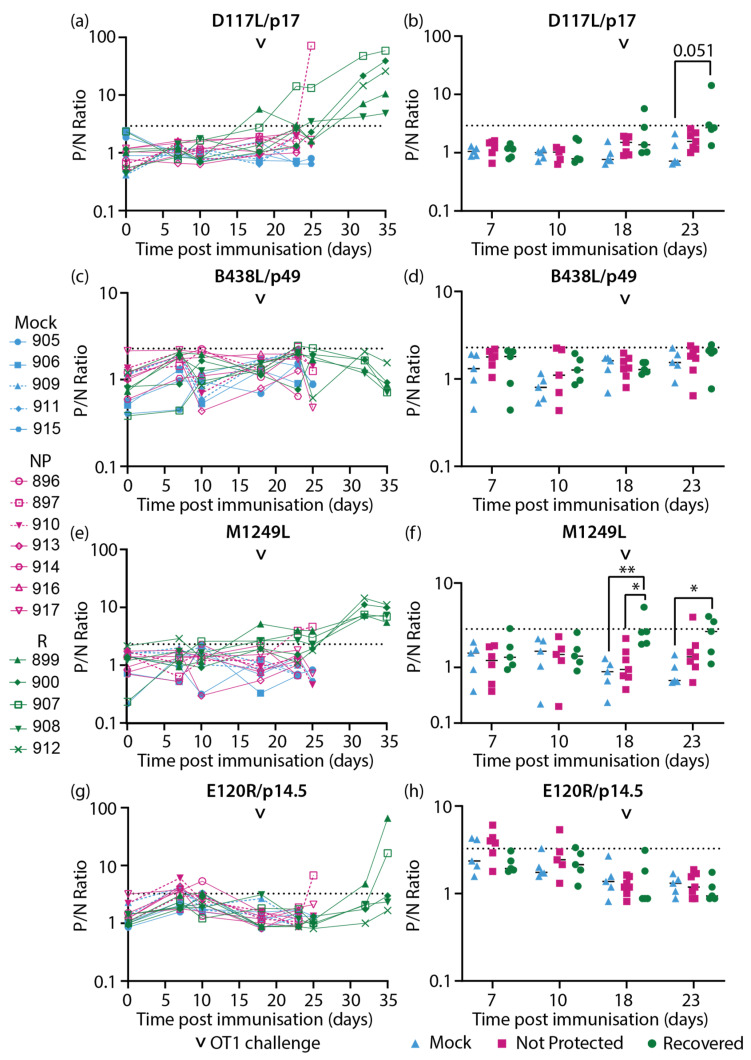
Longitudinal antibody responses of inbred Babraham pigs to ASFV recombinant capsid proteins (**a**,**b**) D117L/p17, (**c**,**d**) B438L/p49, (**e**,**f**) M1249L, and (**g**,**h**) E120R/p14.5 detected with antigen-specific LACAs at selected dpi. (**a**,**c**,**e**,**g**) Antigen-specific antibody kinetics of each animal are plotted. (**b**,**d**,**f**,**h**) Antibody responses consolidated as a group at each relevant time point are plotted. Only time points where there were four data points or more were included in this analysis. Lines indicate the mean. Each data point corresponds to a single animal. The point of challenge with virulent OURT88/1 (OT1, 18 dpi) is denoted by the arrowhead in each graph. P/N Ratio: ratio of luciferase activity of each sample to the luciferase activity of the negative control. Dashed line indicates the cutoff determined from the mean and 3 × standard deviation of all negative sera samples in each experiment. Blue, Mock: mock control animals immunized with PBS, Pink, NP: OURT88/3 immunized animals that were not protected from OURT88/1, Green, R: OURT88/3 immunized animals that recovered from OURT88/1. * *p* < 0.05, ** *p* < 0.01, mixed effects model.

Sera samples collected from inbred Babraham animals [50] (Figure 1) were assayed with the CP204L/p30 and ASFV capsid protein-specific LACAs. Antigen-specific antibody responses were detected against CP204L/p30 (Figure 4a,b), the combination of B646L/p72-B602L (Figure 4c,d), B646L/p72 (Figure 4e,f), the chaperone B602L (Figure 4g,h), D117L/p17 (Figure 5a,b), M1249L (Figure 5e,f), and E120R/p14.5 (Figure 5g,h). Antibody responses for B438L (Figure 5c,d) were not observed, potentially due to lower expression of B438L, and low or non-immunogenicity of this protein. Sample timepoints are designated using “days post immunization” (dpi) to indicate the time following immunization and “days post challenge” (dpc) to denote the time elapsed after the virulent ASFV challenge.

CP204L/p30 antibodies were detected in most of the animals by 10 days post immunization (10 dpi, Figure 4a) and, interestingly, were higher in the recovered group in comparison to the not-protected group at this time point (Figure 4b). However, by the day of challenge, similar levels of CP204L/p30 antibodies were observed in both recovered and not-protected animals (18 dpi, Figure 4a,b) and at subsequent timepoints. Antibodies that recognized B646L/p72 when co-expressed with B602L (Figure 4d) as well as B602L itself (Figure 4h) were detected at challenge (18 dpi) in most of the animals that recovered from OURT88/1 challenge. In contrast, animals that were not protected had lower levels of B646L/p72 and B602L antibodies (Figure 4d) at challenge. By the time the non-protected animals reached their humane endpoints, only one animal (pig 897) had an observable increase in antibodies against B646L/p72-B602L and B602L (Figure 4c and Figure 4g, respectively). Antibodies targeting B602L-independent B646L/p72 conformational epitopes were not quantifiable in the assay with B646L/p72 until 32 dpi in recovered animals (Figure 4e). In most of the animals that recovered from challenge, anti-B646L/p72 and B602L antibody levels increased between 25 and 32 dpi (7 and 14 days post challenge, dpc, Figure 4e,g) and plateaued at 32 dpi/14 dpc. These results indicated that recovery from OURT88/1 infection may be associated with anti-B646L/p72-B602L and B602L antibody levels at the point of challenge.

In recovered animals, antibodies targeting D117L/p17 (Figure 5a) and M1249L (Figure 5e) were generally observed to increase by 32 dpi/14 dpc and stabilize between 32 dpi/14 dpc and 35 dpi/17 dpc. Increases in anti-E120R/p14.5 antibodies were only detected in two animals in the recovered group (pigs 899 and 907, Figure 5g), and this was only evident at termination on 35 dpi/17 dpc. In the non-protected group, pig 897 had increased antibody levels against D117/p17 (Figure 5a), M1249L (Figure 5e), and E120R/p14.5 (Figure 5g) at the point of termination (24 dpi/6 dpc), in contrast to the other animals that were not protected.

**Figure 6:**
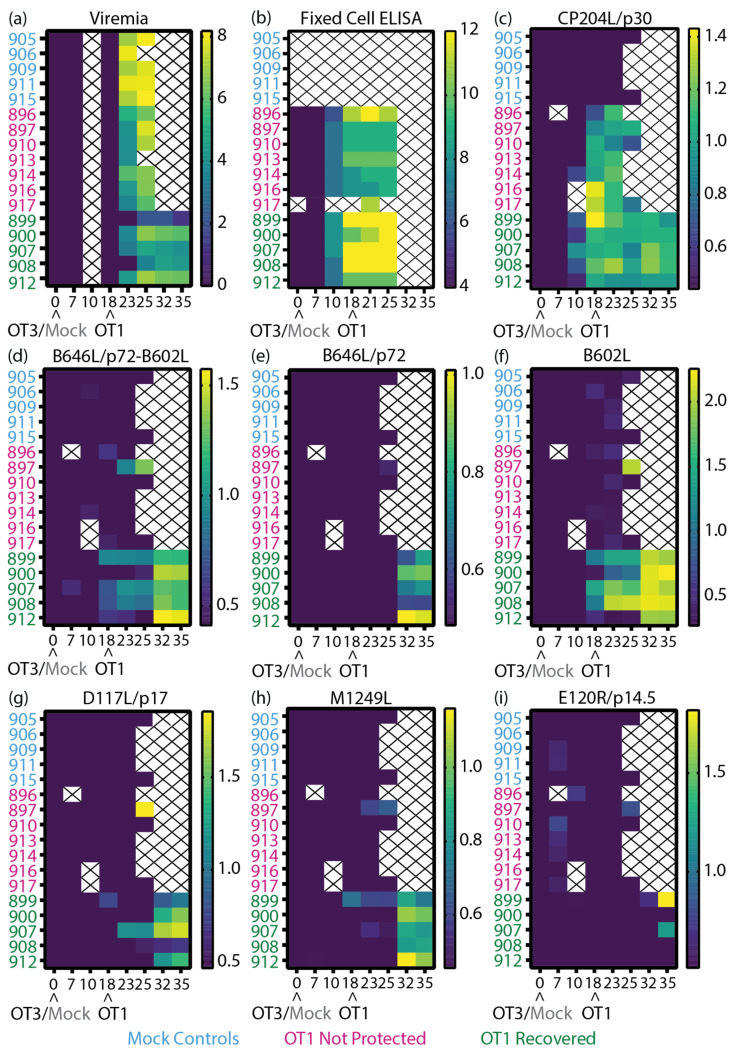
Heatmaps of the virological and immunological parameters of inbred Babraham animals. (**a**,**b**) Data reported previously [50], (**a**) viremia, (**b**) anti-ASFV antibody titer as determined by fixed-cell ELISA on BA71V-infected Vero cells [50], and (**c**–**i**) recombinant ASFV protein-specific LACAs targeting (**c**) CP204L/p30, (**d**) B646L/p72 co-expressed with B602L chaperone, (**e**) B646L/p72, (**f**) B602L chaperone, (**g**) D117L/p17, (**h**) M1249L, and (**i**) E120R/p14.5. Data plotted as (**a**) Log10 genome copy numbers/mL, (**b**) Log2 antibody titer, and (**c**–**i**) Log10 of P/N ratio. Each row denotes the responses of a single animal. The negative cutoff for each protein-specific LACA was determined from the mean and 3x standard deviation of all negative sera samples in each experiment. Animal numbers are indicated on the *y*-axis and the time post-immunization is denoted on the *x*-axis. Crosses indicate samples that were not available for analysis. Arrowheads denote the immunization and ASFV challenge time points. Blue, Mock: mock control animals immunized with PBS, Pink, NP: OURT88/3 immunized animals that were not protected from OURT88/1, Green, R: OURT88/3 immunized animals that recovered from OURT88/1.

To relate the antigen-specific antibody responses measured in this study with the clinical (Supplementary Figure S5), virological (Figure 6a), and anti-ASFV antibody (Figure 6b) data previously collected [50], heatmaps of the different data sets were plotted (Figure 6) to determine if trends were observable. In the previous study [50], antibody levels against the highly immunogenic CP204L/p30 were detected with a commercially available blocking ELISA. Here, the CP204L/p30 LACA (Figure 6c) broadly confirmed our previous CP204L/p30 ELISA results [50]. The higher anti-ASFV antibody responses in recovered animals measured with the fixed-cell ELISA (Figure 6b) generally corresponded to the kinetics observed with B646L/p72-B602L (Figure 6d) and B602L (Figure 6f).

Of all the proteins tested, CP204L/p30 had the strongest responses in the not-protected group (Figure 6c). Within the animals that were not protected, pig 897 raised antibodies against B646L/p72-B602L (Figure 6d), B602L (Figure 6f), D117L/p17 (Figure 6g), M1249L (Figure 6h), and E120R/p14.5 (Figure 6i) by the time it was culled, and its end point temperature (40.6 °C, Supplementary Figure S5b) was the lowest within its group. Pig 896 had higher ASFV antibody titers in the fixed-cell ELISA (Figure 6b), but antibodies to the panel of antigens (Figure 6d–i) were not detected in this animal, so there may be antibodies to other antigens besides CP204L/p30 (Figure 6c) that contribute to the response measured by the fixed-cell ELISA in this animal.

Amongst the recovered animals, pigs 900 and 912 had higher viremia on 25 dpi/7 dpc (Figure 6a) and displayed clinical signs around the same time as the animals that were not protected (Supplementary Figure S5a,b). Both 900 and 912 displayed the lowest amount of anti-ASFV (Figure 6b), B646L/p72-B602L (Figure 6d), and B602L (Figure 6f) antibodies within the recovered animals on the day of challenge (18 dpi). As part of the secondary humoral response from pig 900, increased ASFV antibody titers were measured from 21 dpi/3 dpc (Figure 6b), possibly with contribution from anti-B602L antibodies (Figure 6f) due to the similar kinetics. Antibodies targeting the ASFV capsid proteins assessed in this panel were not detected in the sera of pig 912 until 32 dpi/14 dpc, so antibodies against other ASFV antigens may play a contributing role in the recovery of this animal. By the end of the study, both animals displayed the highest viremia within the group, which corresponded with higher B646L/p72-B602L (Figure 6d), B646L/p72 (Figure 6e) and M1249L (Figure 6h) antibody levels.

Pigs 899 and 908 displayed delayed clinical signs (Supplementary Figure S5a,b) and viremia (Figure 6a), while pig 907 showed milder clinical signs (Supplementary Figure S5a). All three animals produced detectable levels of B646L/p72-B602L (Figure 6d) and B602L (Figure 6f) antibodies from 18 dpi/0 dpc until the end of the study, which may have contributed to the delay of viremia and/or milder clinical signs. In contrast to the rest of the group, the levels of D117L/p17 antibody increased in animal 907 after challenge (Figure 6g), while animal 908 maintained higher levels of B602L (Figure 6f) antibodies. Pig 899 had the lowest viremia detected in the study, and this was complemented by higher CP204L and B646L/p72-B602L antibody levels (in comparison to the rest of the group, Figure 6c,d), detectable antibody levels to D117L/p17 (Figure 6g), and production of anti-M1249L antibodies (Figure 6h) on 18 dpi/0 dpc. After peak viremia (25–32 dpi, Figure 6a), pig 899 had high levels of B602L (Figure 6f) and E120R/p14.5 (Figure 6i) antibodies.

### 3.2. Antigen-Specific Antibody Respones of Outbred Domestic Pigs

**Figure 7:**
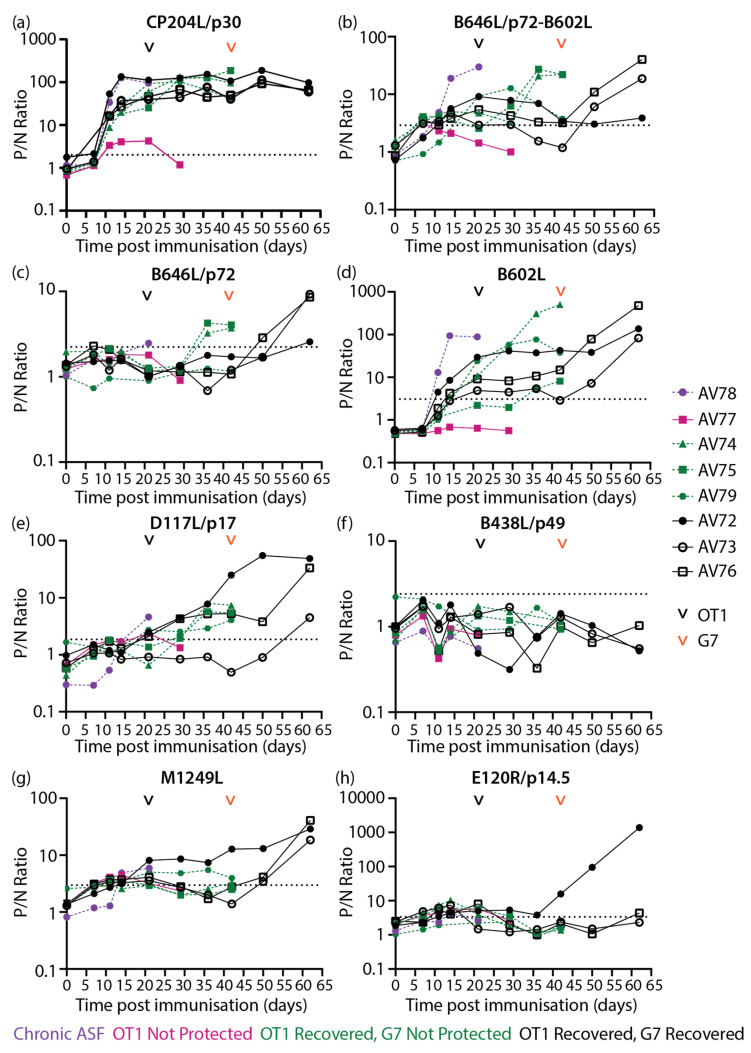
Longitudinal antibody responses of outbred domestic pigs to ASFV recombinant proteins (**a**) CP204L/p30, (**b**) B646L/p72 co-expressed with chaperone B602L, (**c**) B646L/p72, (**d**) B602L chaperone, (**e**) D117L/p17, (**f**) B438L/p49, (**g**) M1249L, and (**h**) E120R/p14.5 detected with protein-specific LACAs at selected dpi. Antigen-specific antibody kinetics of each animal are plotted. Each data point corresponds to a single animal. The point of challenge with virulent OURT88/1 (OT1, 21 dpi) and virulent Georgia 2007/1 (G7, 42 dpi) is denoted by the black and orange arrowheads, respectively. P/N Ratio: ratio of luciferase activity of each sample to the luciferase activity of the negative control. Dashed line indicates the cutoff determined from the mean and 3x standard deviation of all negative sera samples in each experiment. Purple: OURT88/3 immunized animal that suffered from chronic ASF, Pink: OURT88/3 immunized animal that was not protected from OURT88/1, Green: OURT88/3 immunized animals that recovered from OURT88/1, but were not protected from Georgia 2007/1, Black: OURT88/3 immunized animals that recovered from OURT88/1 and Georgia 2007/1.

Sera samples collected from outbred domestic animals [50] (Figure 2) were also subjected to LACAs with ASFV capsid proteins and CP204L/p30. Antibody responses were detected against CP204L/p30 (Figure 7a), B646L/p72-B602L (Figure 7b), B646L/p72 (Figure 7c), B602L (Figure 7d), D117L/p17 (Figure 7e), M1249L (Figure 7g), and E120R/p14.5 (Figure 7h). Antibodies were not quantifiable with the B438L/p49 assay (Figure 7f), similarly to observations with inbred Babraham animals (Figure 5c).

High levels of CP204L/p30 antibodies were detected in most animals from 11 dpi and remained high throughout the study (Figure 7a), broadly confirming previous CP204L/p30 blocking ELISA results [50]. The differences in antibody levels identified with the B646L/p72-B602L assay (Figure 7b) in comparison to the B602L independent B646L/p72 assay (Figure 7c) were largely similar to results observed with inbred Babraham animals (Figure 4c,e), where higher levels of B646L/p72 antibodies were detected when co-expressed with B602L. B646L/p72-B602L (Figure 7b) and B602L (Figure 7d) antibody levels increased strongly in AV78, which developed chronic ASF, and also in two of the animals that did not survive challenge with Georgia 2007/1 from 36 dpi/15 dpc OT1 (days post challenge with OURT88/1). Antibodies against B602L-independent epitopes of B646L/p72 (Figure 7c) also increased after challenge in pigs AV74 and AV75 in a similar pattern to the B602L-dependent assay (Figure 7b). In animals that survived challenge with Georgia 2007/1, B646L/p72-B602L (Figure 7b) and B602L (Figure 7d) antibodies were measured on 21 dpi/0 dpc OT1. Higher B602L antibody levels (Figure 7d) were generated in all animals within this group from 50 dpi/8 dpc G7 (days post challenge with Georgia 2007/1) onwards, while elevations in B646L/p72-B602L (Figure 7b) and B646L/p72 (Figure 7c) antibodies were only observed in two animals within the group.

Low levels of D117L/p17 antibodies (Figure 7e) were measured in most of the animals that survived the OURT88/1 challenge and in the lone animal with chronic ASF. These levels increased in two of the animals that survived to the end of the study after the challenge with Georgia 2007/1. Likewise, M1249L antibodies increased in animals that recovered from Georgia 2007/1 (Figure 7g). Low levels of M1249L antibodies were detected in the animal that developed chronic ASF and in one of the animals that did not survive the Georgia 2007/1 challenge (Figure 7g). High levels of E120R/p14.5 antibodies (Figure 7h) were produced in one of the animals that survived till the end of the study upon challenge with Georgia 2007/1 (42 dpi).

**Figure 8:**
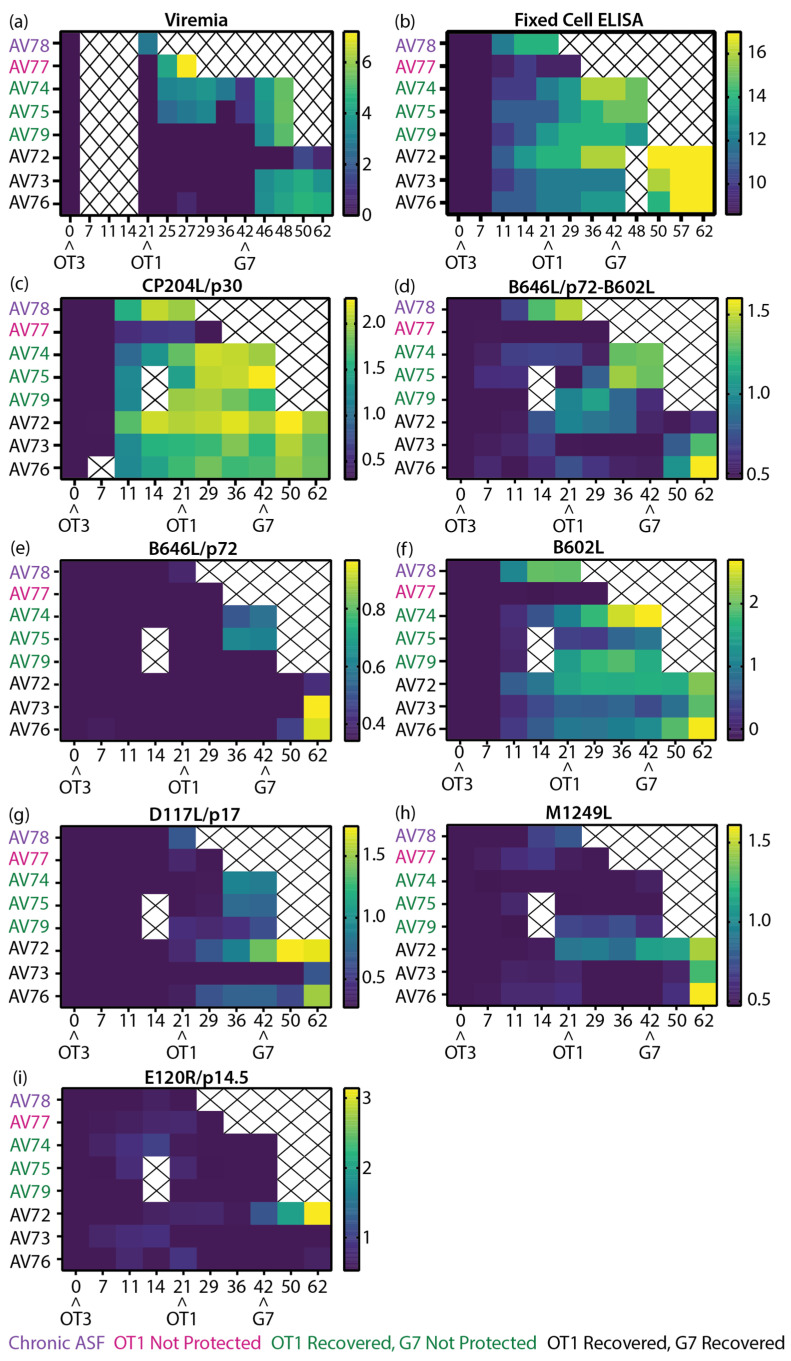
Heatmaps of the virological and immunological parameters of outbred domestic animals. (**a**,**b**) Data collected in previous study [50], (**a**) viremia, (**b**) anti-ASFV antibody titer as determined by fixed-cell ELISA on BA71V infected Vero cells [50], and (**c**–**i**) recombinant ASFV protein-specific LACAs targeting (**c**) CP204L/p30, (**d**) B646L/p72 co-expressed with B602L chaperone, (**e**) B646L/p72, (**f**) B602L chaperone, (**g**) D117L/p17, (**h**) M1249L, and (**i**) E120R/p14.5. Data plotted as (**a**) Log10 genome copy numbers/mL, (**b**) Log2 antibody titer, and (**c**–**i**) Log10 of P/N ratio. Each row denotes the responses of a single animal. The negative cutoff for each protein-specific LACA was determined from the mean and 3x standard deviation of all negative sera samples in each experiment. Animal numbers are indicated on the *y*-axis and the time post-immunization is denoted on the *x*-axis. Crosses indicate samples that were not available for analysis. Arrowheads denote the immunization and ASFV challenge time points. Purple: OURT88/3 immunized animals that suffered from chronic ASF; Pink: OURT88/3 immunized animals that were not protected from OURT88/1; Green: OURT88/3 immunized animals that recovered from OURT88/1 but were not protected from Georgia 2007/1; Black: OURT88/3 immunized animals that recovered from OURT88/1 and Georgia 2007/1.

To facilitate a comparison of the clinical (Supplementary Figure S6), virological (Figure 8a), and antibody (Figure 8b) data from the previous study [50] to the results in this work, heatmaps of all relevant data sets were plotted (Figure 8). Similar to the inbred Babraham sera samples, samples from this experiment were subjected to LACA analysis with Nluc-tagged CP204L/p30 (Figure 8c). All animals produced CP204L/p30 antibodies by 11 dpi and antibody levels remained stable thereafter for most of the animals. AV78 developed chronic ASF after immunization with low-virulent OURT88/3 and this was accompanied by an increase in ASFV antibodies at 11 dpi (Figure 8b) and detectable viremia at 21 dpi/0 dpc OT1 (Figure 8a). Results from the LACAs indicated that antibodies targeting CP204L/p30 (Figure 8c), B646L/p72-B602L (Figure 8d), and B602L (Figure 8f) probably contributed to the increase in antibody titers in this animal. Of the animals that were challenged with OURT88/1, AV77 was the only animal that did not survive this challenge, and this can be attributed to the general poor immunological response of this animal. It had poor cellular responses, as previously reported [50], and here it demonstrated little to no antibody responses to CP204L (Figure 8c) and any of the ASFV capsid antigens tested (Figure 8d–i). The absence of B646L/p72-B602L and B602L antibodies in this animal (Figure 8d,f) corresponded to observations in inbred Babraham animals, where B646L/p72-B602L (Figure 6d) and B602L (Figure 6f) antibodies were associated with recovery from OURT88/1 infection.

The fixed-cell ELISA results (Figure 8b) demonstrated the heterogeneity in ASFV antibody responses amongst the animals that survived the OURT88/1 challenge. Of these six animals, two developed viremia (Figure 8a) and mild clinical signs that resolved quickly (Supplementary Figure S7). Differences between the antibody responses of the two animals (AV74 and AV75) and the rest of the animals that survived could not be identified between 21 dpi/0 dpc OT1 and 29 dpi/8 dpc OT1 with fixed-cell ELISA (Figure 8b), but B646L/p72-B602L (Figure 8d), B646L/p72 (Figure 8e), and B602L (only in AV74, Figure 8f) were observed to increase to higher levels in comparison to the other survivors, and this increase was detected as viremia was decreasing, corresponding to trends observed with viremic Babraham animals (Figure 6d–f). Elevation of D117L/p17 antibodies was measured in all but one (AV73) of the survivors after the OURT88/1 challenge; D117L/p17 antibodies were detected in AV73 after the Georgia 2007/1 challenge (Figure 8g).

The expression of B646L/p72-B602L (Figure 8d), B646L/p72 (Figure 8e), B602L (Figure 8f), and D117L/p17 (Figure 8g) antibodies did not protect three of the animals (AV74, AV75, and AV79) from the Georgia 2007/1 challenge. From the current panel of ASFV capsid antigens, there are no clear differences between the animals that did and did not recover. Similar to animals that developed viremia after the OURT88/1 challenge, the two animals that developed moderate viremia after the Georgia 2007/1 infection and survived to the end of the study also had increased levels of B646L/p72-B602L (Figure 8d), B646L/p72 (Figure 8e), and B602L (only in AV76, Figure 8f) antibodies after peak viremia was reached (Figure 8a).

Interestingly, of the six animals challenged with Georgia 2007/1, pig AV72 had the lowest viremia and displayed delayed clinical signs (Supplementary Figure S6). These were associated with the appearance of D117L/p17, M1249L, and E120R/p14.5 antibodies. AV72 expressed D117L/p17 antibodies (albeit at low levels, Figure 8g) as early as 21 dpi/0 dpc OT1. By the time it received Georgia 2007/1, it had the highest level of D117L/p17 antibodies amongst the challenged animals. Similar antibody kinetics were observed with M1249L (Figure 8h). Furthermore, AV72 was the only animal to express detectable amounts of E120R/p14.5 at 42 dpi (day of the Georgia 2007/1 challenge, Figure 8i) and to increase production of E120R/p14.5 antibodies in a tertiary humoral immune response.

## 4. Discussion

In this study, we sought to resolve the ASFV antigen-specific antibody responses of our previous study with inbred Babraham animals and outbred domestic animals that were immunized with low-virulent OURT88/3 for antigen discovery purposes. Using a panel of modified LACAs targeting specific recombinant ASFV capsid proteins and the known chaperone B602L, we were able to characterize a small section of the complex humoral responses in the animals against ASFV capsid proteins.

The earliest antibody responses identified in animals were targeting CP204L/p30 (Figure 4 and Figure 7a), B602L (Figure 4 and Figure 7d), and, to some extent, B646L/p72 (when expressed in combination with B602L, Figure 4c and Figure 7b), and these antibodies potentially contributed to titers observed at 11 dpi in the fixed-cell ELISA data collected previously [50] (Figure 6b and Figure 8b). Of the antigen-specific antibody responses probed, CP204L/p30-specific antibody levels were the highest in the Babraham animals that were not protected (Figure 4a and Figure 6c), confirming the strong immunogenicity of CP204L/p30 [22,55,56,57]. This is in contrast to the other antigens in the panel (Figure 6) suggesting a contributory role of anti-CP204L/p30 antibodies to the fixed-cell ELISA antibody titers observed in these animals (Figure 6b). CP204L/p30 antibody levels were higher at 10 dpi in Babraham animals that recovered from OURT88/1 in comparison to animals that were not protected (Figure 4b and Figure 6c). Similarly, early expression of CP204L/p30 antibodies was observed in outbred animals that recovered from OURT88/1 challenge (Figure 7a and Figure 8c), indicating that earlier expression of anti-CP204L/p30 antibodies may contribute to the reduction of OURT88/1-induced clinical signs and viremia. Anti-CP204L/p30 antibodies can be detected as early as eight days post-infection [22], and a potential role for CP204L/p30 antibodies in protection against genotype I ASFV has been suggested [56,58].

B438L/p49 was observed to be low or non-immunogenic in both Babrahams and outbred pigs (Figure 5c and Figure 7f), which is consistent with data from Lokhandwala et al. [46]. This could be attributed to improper folding of the antigen—potentially a requirement for co-expression of a chaperone similar to B646L/p72—or the potential location of B438L/p49 in the overall structure of the capsid as resolved by Wang et al. [33] with cryo-electron microscopy methods. They postulated that B438L/p49 is positioned on the inner shell of the capsid vertices, connecting the overlying penton proteins to the inner membrane. It is possible that such a location and potential steric hindrance by penton proteins hinder the development of a strong antibody response to this protein. Furthermore, antibody responses were generally slower to develop against the minor capsid proteins D117L/p17 and M1249L (Figure 6 and Figure 8), possibly due to their frequency and location within the virion.

The animal that developed chronic ASF (AV78, Figure 8), increased production of B602L and B646L/p72-B602L antibodies was evident as the disease progressed. These observations are consistent with the data obtained by Reis et al. [59] in their analysis of antigen-specific antibody responses from animals that developed chronic disease after infection with the low-virulent ASFV/NH/p68. Separately, the presence of B646L/p72 and B602L antibodies (Figure 6 and Figure 8) was associated with recovery from OURT88/1 challenge in both inbred Babrahams and outbred pigs, but these were not sufficient to prevent viremia induced by OURT88/1. Both B646L/p72 and B602L were previously included in a pool of eight antigens that afforded protection from lethal disease following infection with OURT88/1 [14]. Furthermore, both antigens have been components of a pool of adenovirus-vectored antigens in vaccinated farm pigs, which have been shown to impart protection against ASFV [60]. B646L/p72 and B602L antibodies did not correlate with protection against Georgia 2007/1 in outbred animals (Figure 8). Due to the small number of outbred animals and the limited panel of ASFV antigens in this study, it was not possible to differentiate antigen-specific antibody responses between animals that did and did not recover from Georgia 2007/1. However, the observations of antibody responses against M1249L and E120R/p14.5 in recovered animals with reduced clinical signs and viremia after the OURT88/1 and Georgia 2007/1 challenges warrant further investigation.

Similar to our previous findings with regard to the cellular responses of these animals [50], there were marked differences in the antibody responses between Babraham animals and the outbred animals that may account for differences in clinical outcomes. Over half of the immunized Babraham animals were unable to mount a response to B646L/p72-B602L and B602L (Figure 6d,f), despite the ability to develop CP204L/p30 antibodies (Figure 6c). In contrast, the majority of the outbred animals produced antibodies to B646L/p72-B602L and B602L (Figure 8d,f), and the poor outcome of AV77 can be attributed to a generally poor immune response, as observable in its low levels of CP204L/p30 antibodies (Figure 8c), in comparison to the other animals in both experiments. As discussed in Goatley et al. [50], the increased susceptibility of Babraham animals to OURT88/1 following vaccination with OURT88/3 could potentially stem from various factors, including environmental conditions at different breeding sites, or unique host genetics inherent to the Babrahams. Babrahams are a highly inbred line, which might result in a narrower antibody repertoire [61]. Investigations into antibody repertoire variations in humans have revealed associations between immunoglobulin alleles and antibody affinity, as well as their related functions, which in turn influence susceptibility to infections [62,63]. This study focused on antibody responses to ASFV capsid proteins. Hence, it remains plausible that differences in humoral responses to other ASFV proteins also contribute to the divergent susceptibility observed. These factors may collectively contribute to the observed differences in ASFV susceptibility and merit further examination.

Previous results [6,50,64] demonstrated the potential of cross-protection between genotype I and II ASFV strains, and here we sought to identify if genotype I-trained antibody responses would be able to bind to genotype II antigens since most of the capsid proteins are highly conserved with amino acid sequence identities between 93.4 and 99.4%, with the exception of B602L, which has a sequence identity of 78.0% due to the central variable region [40] (Supplementary Figures S7–S13). In both experiments, antibody responses could be detected with the genotype II recombinant proteins, even against B602L, which had the lowest homology between genotypes due to the presence of a central variable region [40] (Supplementary Figure S8). Here, we confirm that cross-reactive antibodies are present for all capsid proteins, apart from B438L, assessed in this work.

Vaccine research efforts have mainly focused on cellular responses [6,11,13] even though robust protection against ASFV involves both humoral and cellular immune pathways [15]. More recently, the action of antibodies induced by live attenuated vaccines has been associated with survival and protection after the virulent Georgia 2007/1 challenge, and the presence of ASFV neutralizing antibodies [18]—a point of contention for decades [19]—was described. Our work presented here demonstrates the contributions of antibody responses to protection from fatal disease and identifies a subset of the contributing antigens in inbred animals and, to a lesser extent, outbred pigs. The tools presented here expand the ability to decipher the antibody responses of non-protected and convalescent animals after immunization and the ASFV challenge. Wider screening of animals that respond differently would enable the identification of antigen-specific antibodies that correlate with protection. These identified antibodies can then be prioritized for further investigations into their effector functions in future studies.

In contrast to antigen-specific ELISAs that have been developed using recombinant ASFV antigens in bacterial [21,22,23,25,26] or insect cells [26,65,66], the assays described here use recombinant proteins produced in mammalian cells, similar to the EP402R/CD2v ELISA developed by Lv et al. [24] and the LIPS assay developed by Luong et al. to a panel of ASFV antigens (O61R/p12, CP204L/p30, E183L/p54, CP530R/pp62, EP153R/C-type lectin, and EP402R/CD2v) [28] to ensure that the recombinant proteins receive mammalian post-translational modifications resembling their native conformation. The ability to utilize unpurified recombinant proteins in cell lysates for the assay simplifies the process [30,31] and enables the development of antigen discovery assays using antigens that are typically difficult to purify, like membrane-bound proteins or hard-to-express proteins with low yields, which is typical of many ASFV proteins that have immunomodulatory properties. ELISAs remain the gold standard for ASFV serology diagnostics, but the use of LACAs can expedite the identification of potential protective antigens to be incorporated into vaccine candidates.

Unlike LIPS, LACA has a lower dynamic range due to the use of white plates coated with protein A instead of protein A resin, but this setup drastically reduces the cost and requirements for specialized equipment and filter plates. Furthermore, the use of white plates provides the option to use isotype-specific antibodies for antibody capture, as described by Duong et al. in their development of a LACA for antibody responses from chickens [67], since protein A has different affinities for antibodies from different species and subclasses. Further discrimination of antigen-specific antibody responses at the subclass level will help direct studies into other antibody-directed innate effector functions [68] that may contribute to protection.

While improving cellular responses to vaccinations is important for protection against ASFV, increased emphasis needs to be placed on exploring the repertoire of ASFV antigen-specific antibody responses of vaccinated and convalescent animals to identify protective antigens and inform vaccine design for the development of a safe and efficacious ASFV vaccine.

## Data Availability

The data presented in this study are available on FigShare DOI: 10.6084/m9.figshare.22217284.

## Supplementary Materials

The following supporting information can be downloaded at https://www.mdpi.com/article/10.3390/vaccines11101577/s1, Figure S1: Confocal images of cells expressing recombinant ASFV proteins D117L, B438L, E120R and M1249L; Figure S2: Confocal images of cells expressing recombinant ASFV proteins B646L/p72, B602L and Nluc; Figure S3: Western blot detection of recombinant Nluc-tagged ASFV proteins; Figure S4: Determination of B646L/p72 and chaperone B602L transfection ratio; Figure S5: Heatmaps of (a) clinical scores and (b) temperatures of inbred Babraham animals; Figure S6: Heatmaps of (a) clinical scores and (b) temperatures of outbred domestic pigs; Figure S7: Multiple sequence alignment of B646L/p72 amino acid sequences from Georgia2007/1 and OURT88/3; Figure S8: Multiple sequence alignment of B602L amino acid sequences from Georgia2007/1 and OURT88/3; Figure S9: Multiple sequence alignment of D117L/p17 amino acid sequences from Georgia2007/1 and OURT88/3; Figure S10: Multiple sequence alignment of B438L/p49 amino acid sequences from Georgia2007/1 and OURT88/3; Figure S11: Multiple sequence alignment of E120R/p14.5 amino acid sequences from Georgia2007/1 and OURT88/3; Figure S12: Multiple sequence alignment of M1249L amino acid sequences (1–720) from Georgia2007/1 and OURT88/3; Figure S13: Multiple sequence alignment of M1249L amino acid sequences (721–1249) from Georgia2007/1 and OURT88/3; Supplementary Materials and Methods; Table S1: DNA sequences of recombinant Nluc tagged ASFV protein open reading frames; Table S2: DNA sequence of recombinant Nluc with a 5′ multiple cloning site (MCS) containing a *HindIII* (Capitalised) and *AscI* cloning site (underlined).
