# Supplementary material for: Capsid-Specific Antibody Responses of Domestic Pigs Immunized with Low-Virulent African Swine Fever Virus
Source: Vaccines (Basel). 2023 Oct 10;11(10):1577. doi: 10.3390/vaccines11101577 (PMC10611099; doi:10.3390/vaccines11101577)
Supplement: Supplementary file 1 [file vaccines-11-01577-s001.zip › vaccines-2578767-supplementary.pdf]

Capsid Specific Antibody Responses of Domestic Pigs Immunized with Low Virulent African swine fever virus

Priscilla Y. L. Tng [1,*], Laila Al-Adwani [1], Egle Pauletto [1,2], Joshua Hui [1], and Christopher L. Netherton [1,*]

1 The Pirbright Institute, Ash Road, Pirbright, Woking GU24 0NF, UK

2 The Institute of Medical Sciences, Foresterhill, University of Aberdeen, Aberdeen AB25 2ZD, UK

* Correspondence: priscilla.tng@pirbright.com (P.Y.T.); christopher.netherton@pirbright.ac.uk (C.L.N.)

Supplementary Information

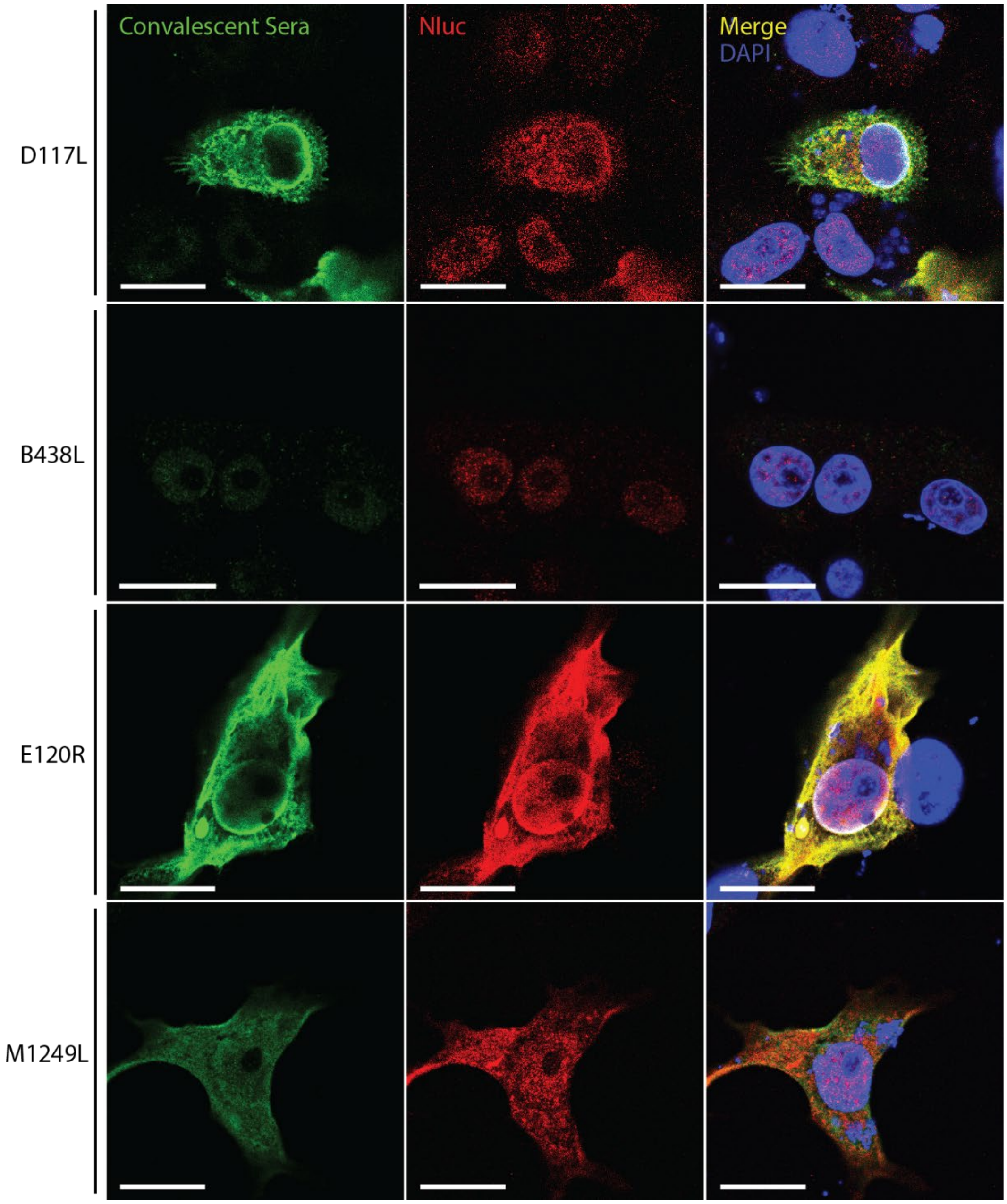

Figure 1. Confocal images of Vero cells expressing recombinant ASFV proteins D117L, B438L, E120R and M1249L. Detection of expression of recombinant nanoluciferase (Nluc)-tagged ASFV proteins in transfected Vero cells was achieved with the monoclonal anti-Nluc antibody (red, Promega) and with convalescent outbred pig sera from our previous study [1] (green). Nuclear DNA was stained with DAPI (blue). White bar denotes 20μm.

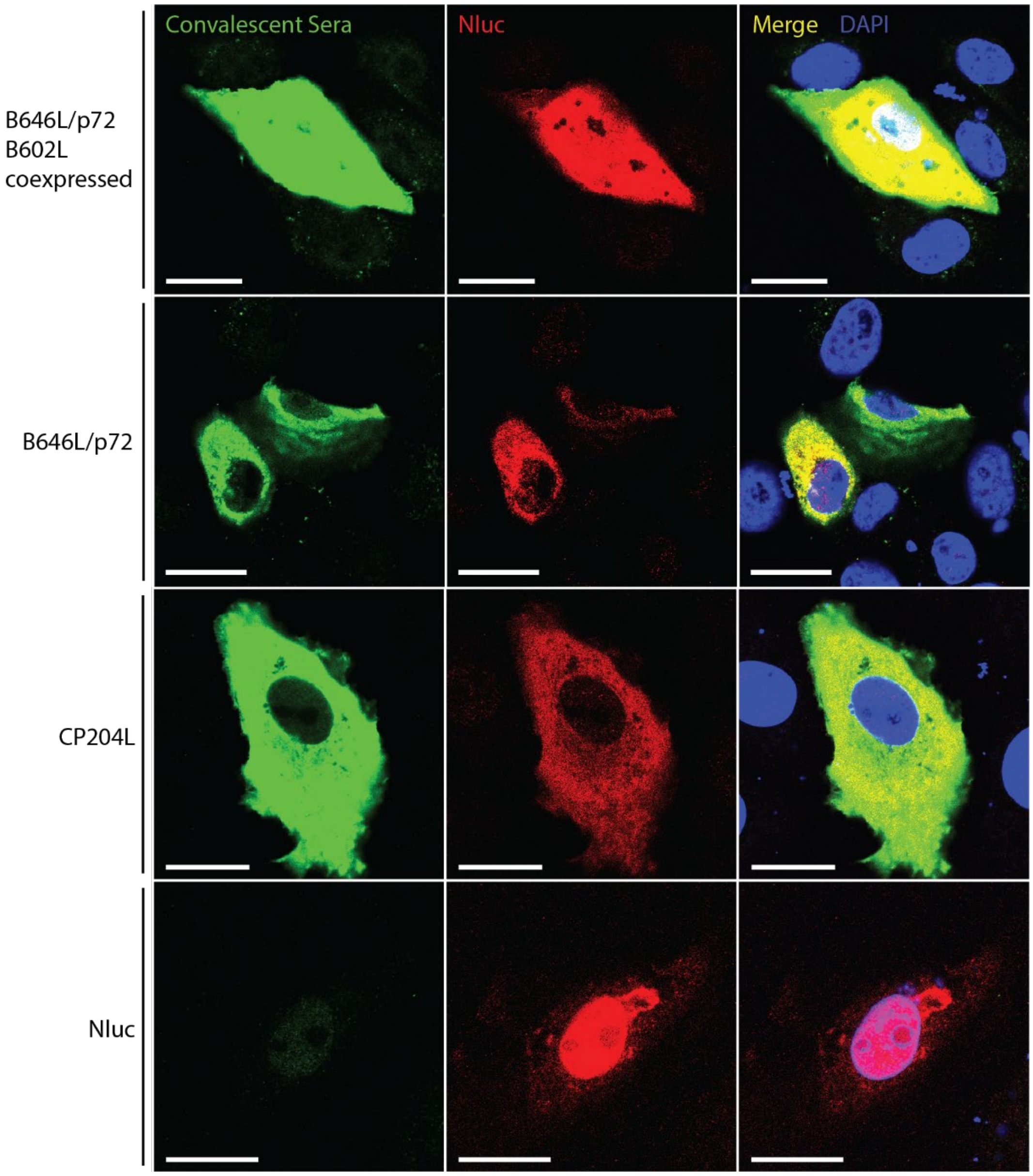

Figure 2. Confocal images of Vero cells expressing recombinant ASFV proteins B646L/p72, B602L, CP204L/p30 and Nluc. B646L/p72 was expressed alone or with chaperone B602L Detection of expression of recombinant nanoluciferase (Nluc)-tagged ASFV proteins in transfected Vero cells was achieved with the monoclonal anti-Nluc antibody (red, Promega) and with convalescent outbred pig sera from our previous study [1] (green). Nuclear DNA was stained with DAPI (blue). White bar denotes 20μm.

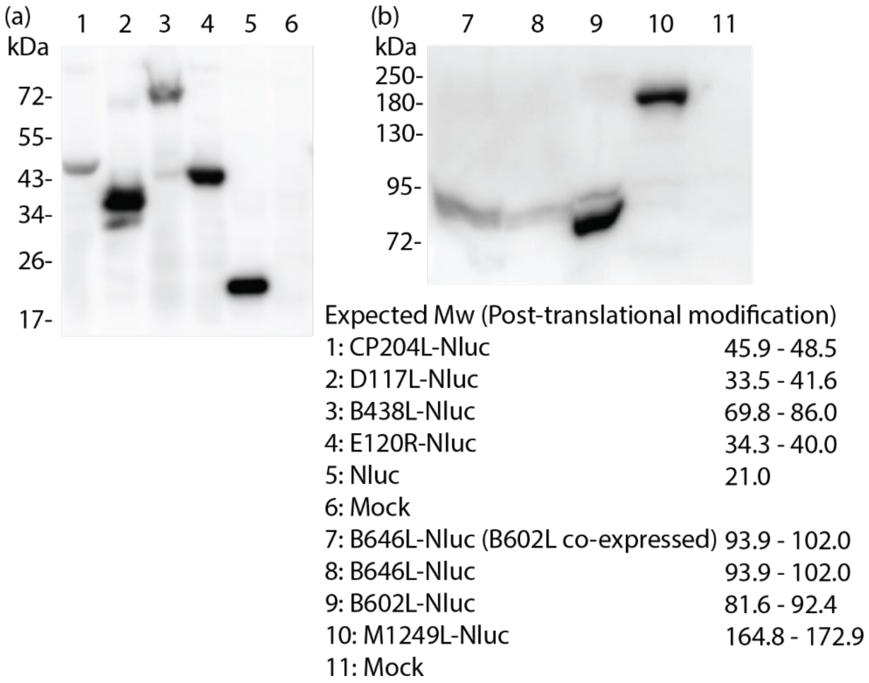

Figure 3. Western blot detection of recombinant Nluc-tagged ASFV proteins. Nluc-tagged ASFV proteins from transfected HEK293T cell lysates were detected with the monoclonal anti-Nluc antibody (Promega). The expected protein molecular weights (Mw) are listed as a range that takes post-translational modifications into account.

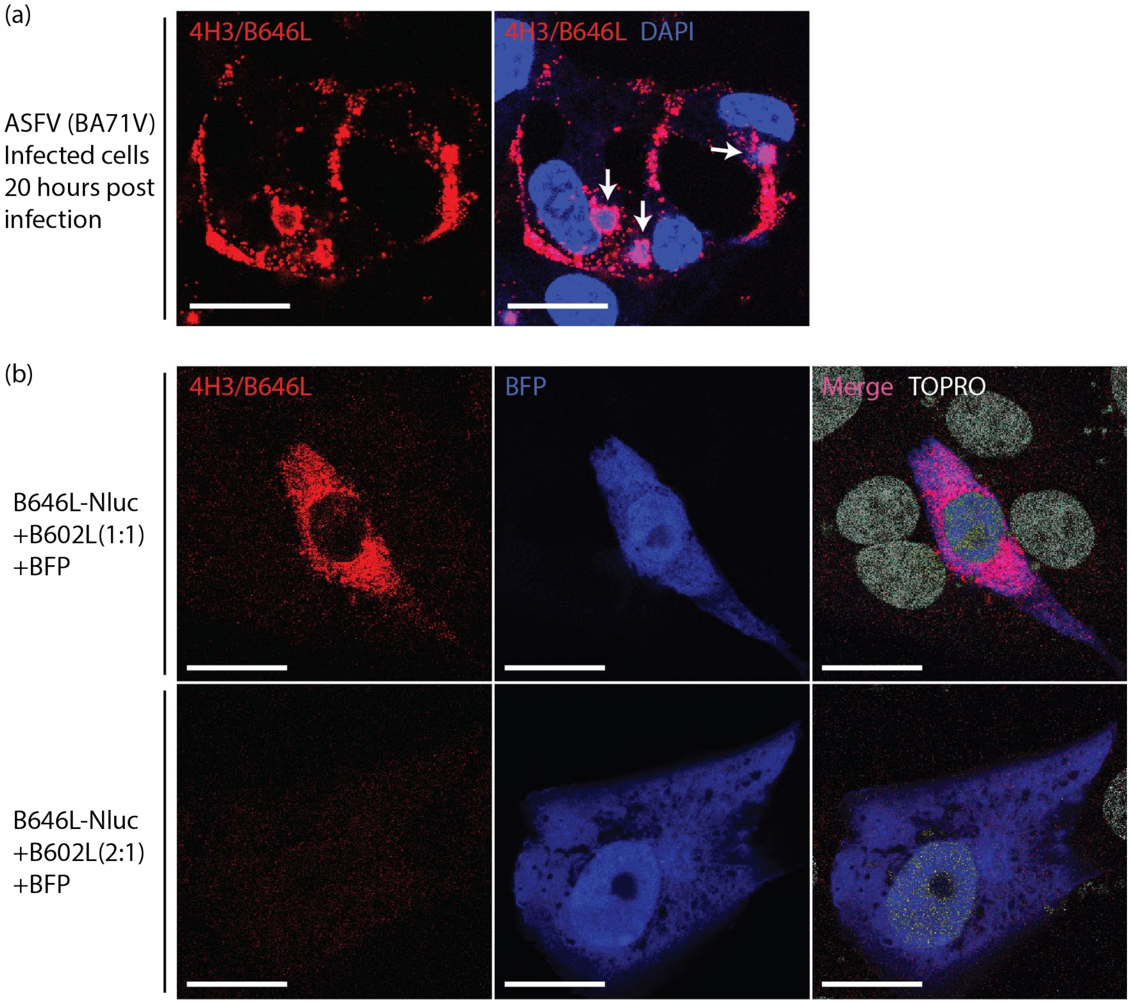

Figure 4. Determination of B646L/p72 and chaperone B602L transfection ratio. (a) ASFV (BA71V) infected Vero cells were used as a positive control for the 4H3 antibody that binds to a conformational epitope on B646L/p72 (red). Nuclear and viral DNA were

stained with DAPI (blue). White arrows indicate ASFV viral factories. (b) Representative confocal images of Vero cells transfected with different ratios of Nluc-tagged B646L/p72 to the chaperone B602L. The amount of B646L-Nluc expressing plasmid was held constant and different amounts of B602L-expressing plasmid were used. A blue fluorescent protein (BFP, blue) expressing plasmid was co-transfected as a transfection control. The 4H3 anti-B646L/p72 antibody (red) was used to detect for expression of recombinant Nluc-tagged B646L/p72 with its native conformation. Nuclear DNA was stained with TOPRO (white). White bar denotes 20μm.

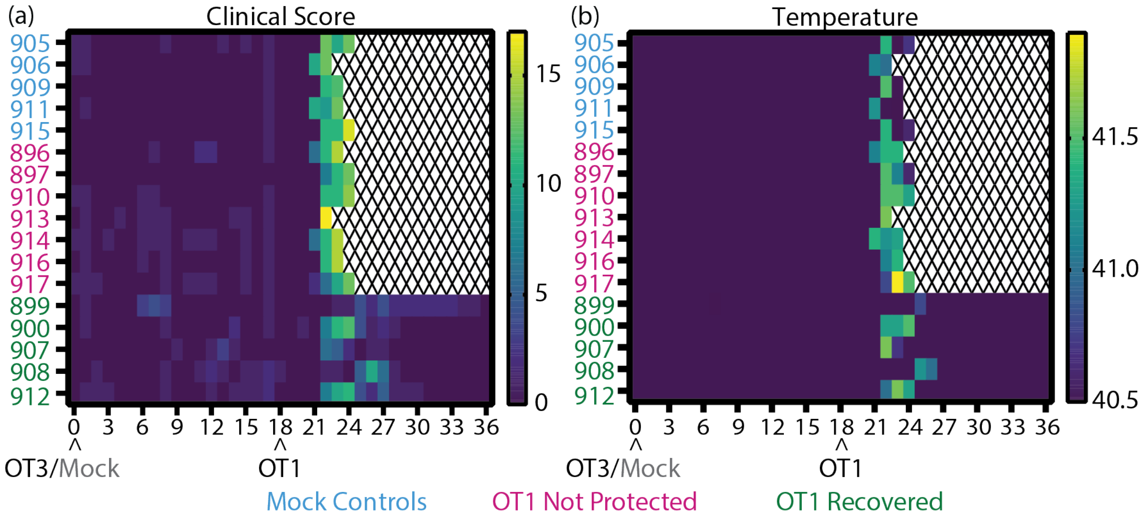

Figure 5. Heatmaps of (a) clinical scores and (b) temperatures of inbred Babraham animals. Data was obtained from our previous study [1]. (b) Data plotted as °C. The negative cutoff for temperature was set at 40.5°C as this is highest value that does not count towards a humane endpoint [1]. Each row denotes the responses of a single animal. Crosses indicate data that were not available for analysis. Arrowheads denote the immunization and ASFV challenge time points. Blue, Mock: mock control animals immunized with PBS, Pink, NP: OURT88/3 immunized animals that were not protected from OURT88/1, Green, R: OURT88/3 immunized animals that recovered from OURT88/1.

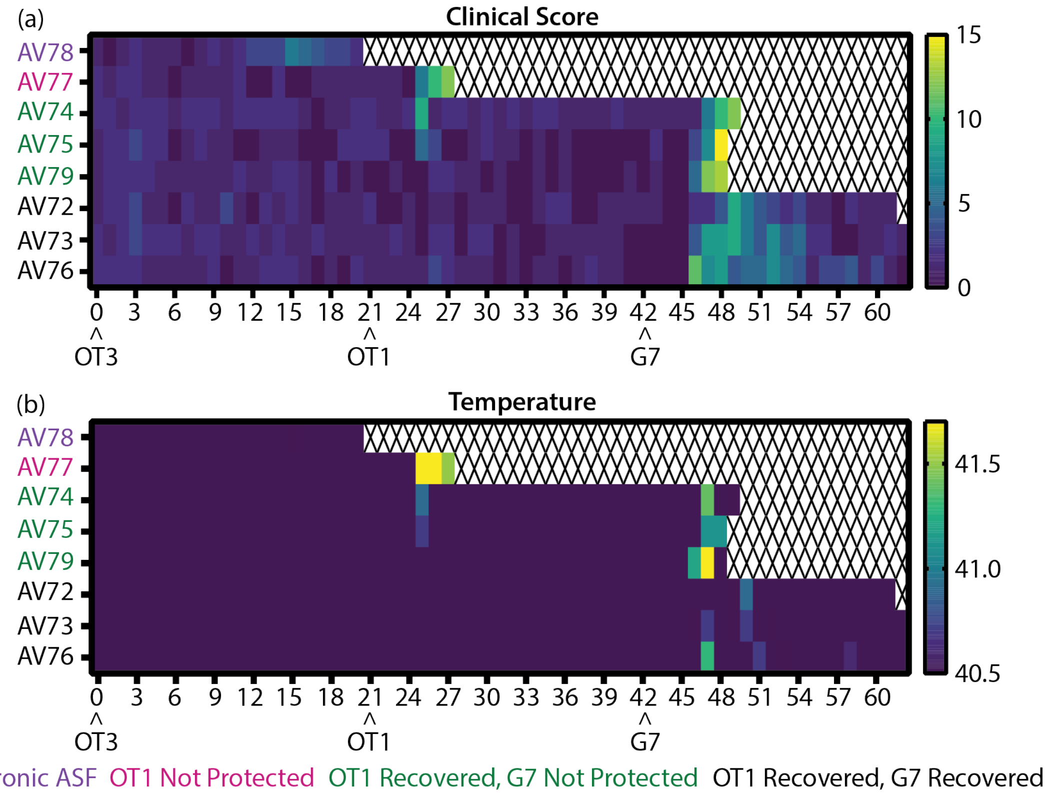

Figure 6. Heatmaps of (a) clinical scores and (b) temperatures of outbred domestic pigs. Data was obtained from our previous study [1]. (b) Data plotted as °C. The negative cutoff for temperature was set at 40.5°C as this is highest value that does not count

towards a humane endpoint [1]. Each row denotes the responses of a single animal. Crosses indicate data that were not available for analysis. Arrowheads denote the immunization and ASFV challenge time points. Purple: OURT88/3 immunized animal that suffered from chronic ASF, Pink: OURT88/3 immunized animal that was not protected from OURT88/1, Green: OURT88/3 immunized animals that recovered from OURT88/1, but were not protected from Georgia 2007/1, Black: OURT88/3 immunized animals that recovered from OURT88/1 and Georgia 2007/1.

```
B646L_Georgia2007_1(NC_044959)  MASGGAFCLIANDGKADKIILAQDLLNSRISNIKNVNKSY  40
     B646L_OURT88_3(NC_044957)  MASGGAFCLIANDGKADKIILAQDLLNSRISNIKNVNKSY  40

B646L_Georgia2007_1(NC_044959)  GKPDPEPTLSQIEETHLVHFNAHFKPYVPVGFEYNKVRPH  80
     B646L_OURT88_3(NC_044957)  GKPDPEPTLSQIEETHLVHFNAHFKPYVPVGFEYNKVRPH  80

B646L_Georgia2007_1(NC_044959)  TGTPTLGNKLTFGIPQYGDFFHDMVGHHILGACHSSWQDA  120
     B646L_OURT88_3(NC_044957)  TGTPTLGNKLTFGIPQYGDFFHDMVGHHILGACHSSWQDA  120

B646L_Georgia2007_1(NC_044959)  PIQGTSQMGAHGQLQTFPRNGYDWDNQTPLEGAVYTLVDP  160
     B646L_OURT88_3(NC_044957)  PIQGTAQMGAHGQLQTFPRNGYDWDNQTPLEGAVYTLVDP  160

B646L_Georgia2007_1(NC_044959)  FGRPIVPGTKNAYRNLVYYCEYPGERLYENVRFDVNGNSL  200
     B646L_OURT88_3(NC_044957)  FGRPIVPGTKNAYRNLVYYCEYPGERLYENVRFDVNGNSL  200

B646L_Georgia2007_1(NC_044959)  DEYSSDVTTLVRKFCIPGDKMTGYKHLVGQEVSVEGTSGP  240
     B646L_OURT88_3(NC_044957)  DEYSSDVTTLVRKFCIPGDKMTGYKHLVGQEVSVEGTSGP  240

B646L_Georgia2007_1(NC_044959)  LLCNIHDLHKPHQSKPILTDENDTQRTCSHTNPKFLSQHF  280
     B646L_OURT88_3(NC_044957)  LLCNIHDLHKPHQSKPILTDENDTQRTCSHTNPKFLSQHF  280

B646L_Georgia2007_1(NC_044959)  PENSHNIQTAGKQDITPITDATYLDIRRNVHYSCNGPQTP  320
     B646L_OURT88_3(NC_044957)  PENSHNIQTAGKQDITPITDATYLDIRRNVHYSCNGPQTP  320

B646L_Georgia2007_1(NC_044959)  KYYQPPLALWIKLRFWFNENVNLAIPSVSIPFGERFITIK  360
     B646L_OURT88_3(NC_044957)  KYYQPPLALWIKLRFWFNENVNLAIPSVSIPFGERFITIK  360

B646L_Georgia2007_1(NC_044959)  LASQKDLVNEFPGLFVRQSRFIAGRPSRRNIRFKPWFIPG  400
     B646L_OURT88_3(NC_044957)  LASQKDLVNEFPGLFIRQSRFIPGRPSRRNIRFKPWFIPG  400

B646L_Georgia2007_1(NC_044959)  VINEISLTNNELYINNLFVTPEIHNLFVKRVRFSLIRVHK  440
     B646L_OURT88_3(NC_044957)  VINEISLTNNELYINNLFVTPEIHNLFVKRVRFSLIRVHK  440

B646L_Georgia2007_1(NC_044959)  TQVTHTNNNHHDEKLMSALKWPIEYMFIGLKPTWNISDQN  480
     B646L_OURT88_3(NC_044957)  TQVTHTNNNHHDEKLMSALKWPIEYMFIGLKPTWNISDQN  480

B646L_Georgia2007_1(NC_044959)  PHQHRDWHKFGHVVNAIMQPTHHAEISFQDRDTALPDACS  520
     B646L_OURT88_3(NC_044957)  PHQHRDWHKFGHVVNAIMQPTHHAEISFQDRDTALPDACS  520

B646L_Georgia2007_1(NC_044959)  SISDISPVTYPITLPIIKNISVTAHGINLIDKFPSKFCSS  560
     B646L_OURT88_3(NC_044957)  SISDISPVTYPITLPIIKNISVTAHGINLIDKFPSKFCSS  560

B646L_Georgia2007_1(NC_044959)  YIPFHYGGNAIKTPDDPGAMMITFALKPREEYQPSGHINV  600
     B646L_OURT88_3(NC_044957)  YIPFHYGGNAIKTPDDPGAMMITFALKPREEYQPSGHINV  600

B646L_Georgia2007_1(NC_044959)  SRAREFYISWDTDYVGSITTADLVVSASAINFLLLQNGSA  640
     B646L_OURT88_3(NC_044957)  SRAREFYISWDTDYVGSITTADLVVSASAINFLLLQNGSA  640

B646L_Georgia2007_1(NC_044959)  VLRYST                                    646
     B646L_OURT88_3(NC_044957)  VLRYST                                    646
```

Figure 7. Multiple sequence alignment of B646L/p72 amino acid sequences from Georgia2007/1 and OURT88/3. These sequences have 99.5% identity.

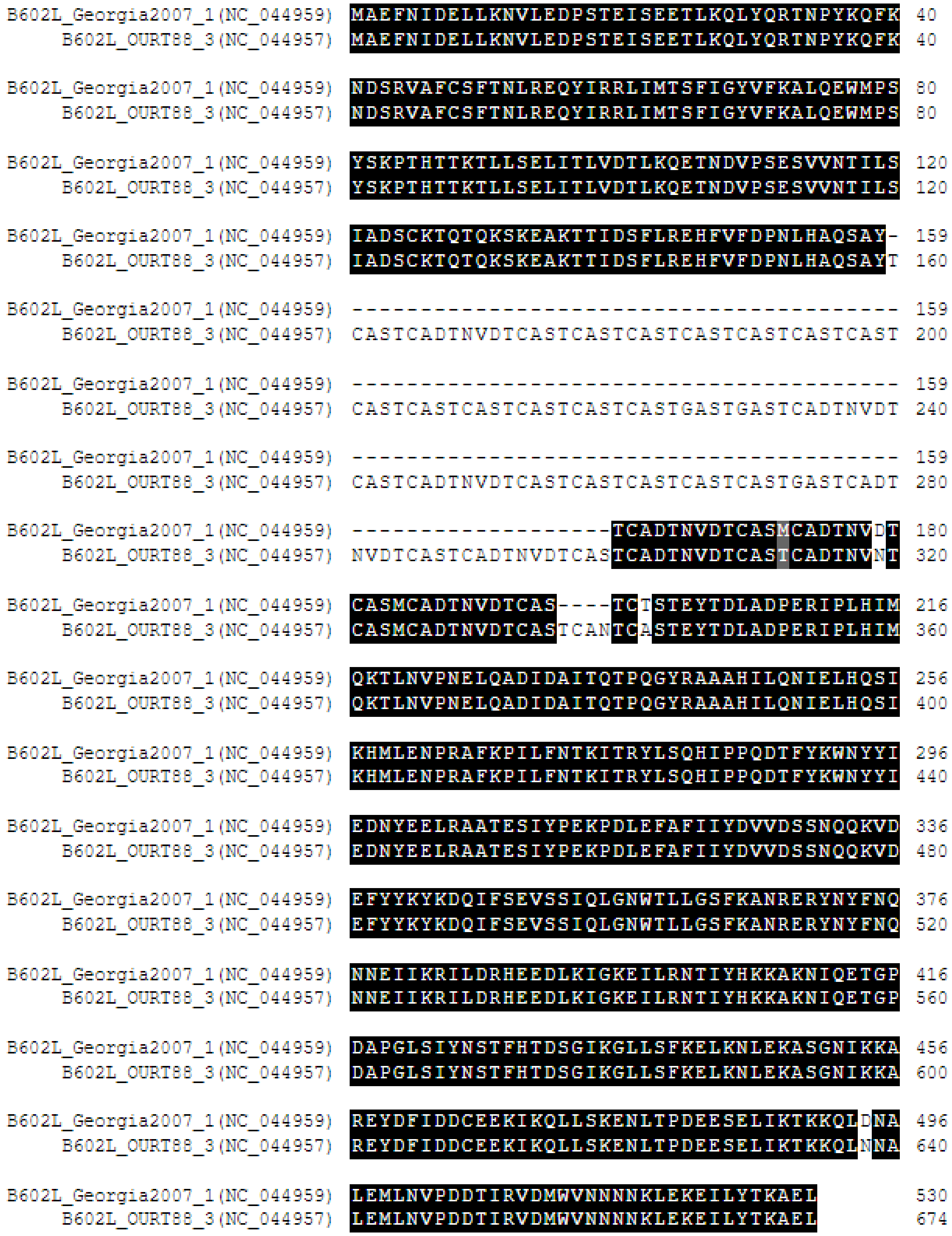

Figure 8. Multiple sequence alignment of B602L amino acid sequences from Georgia2007/1 and OURT88/3. These sequences have 78.0% identity.

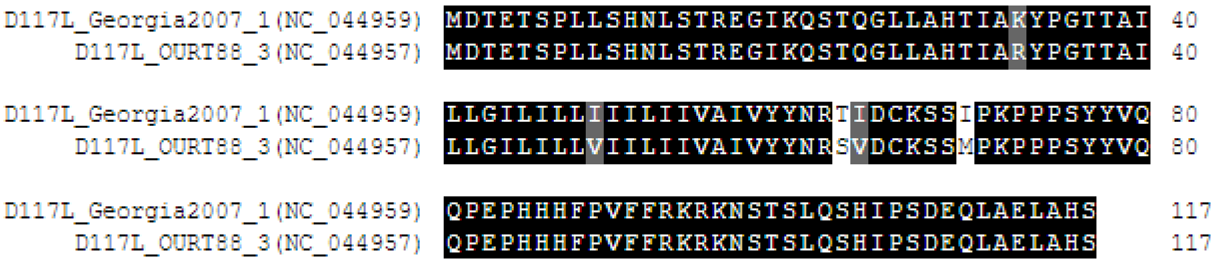

Figure 9. Multiple sequence alignment of D117L/p17 amino acid sequences from Georgia2007/1 and OURT88/3. These sequences have 95.7% identity.

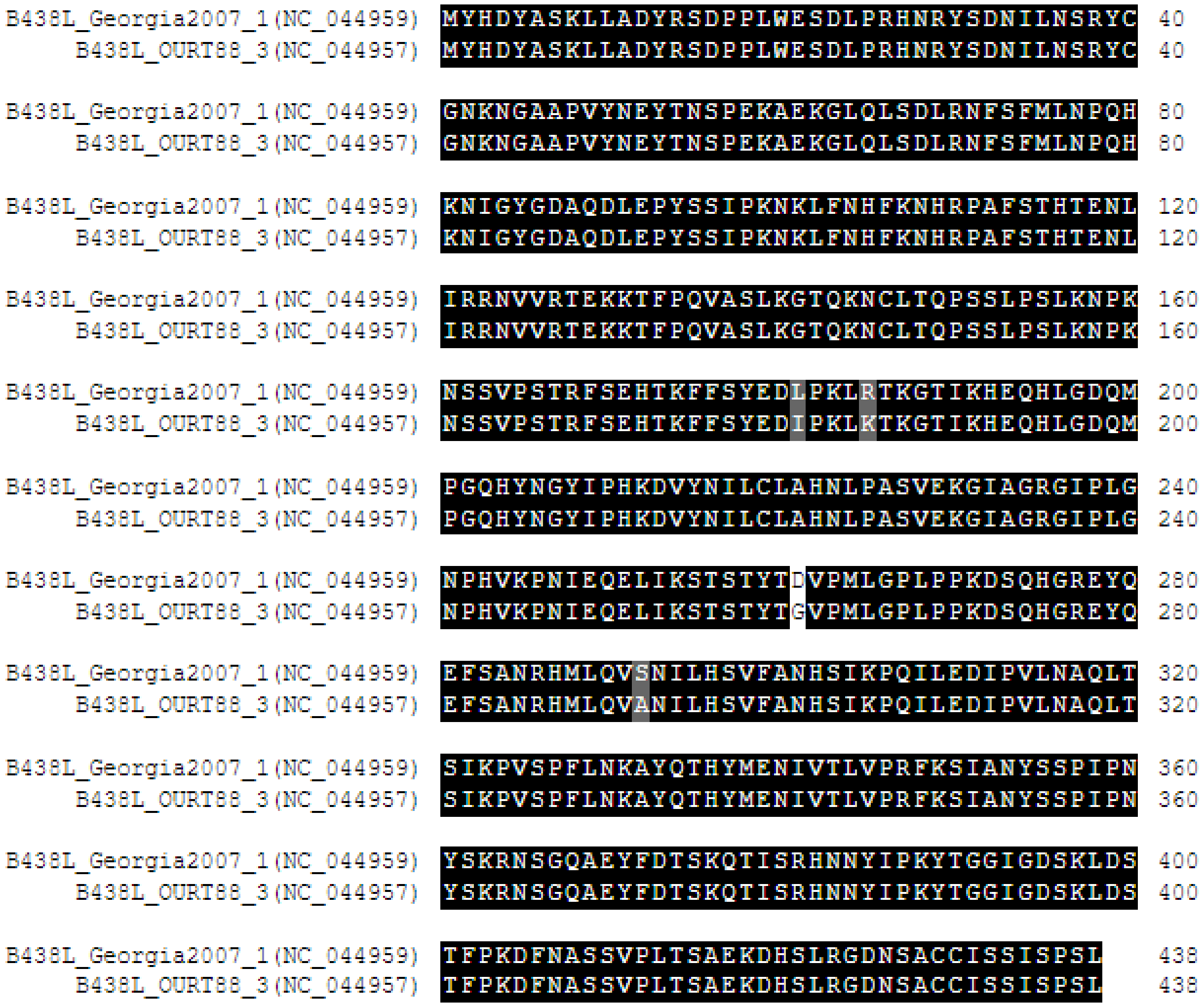

Figure 10. Multiple sequence alignment of B438L/p49 amino acid sequences from Georgia2007/1 and OURT88/3. These sequences have 99.1% identity.

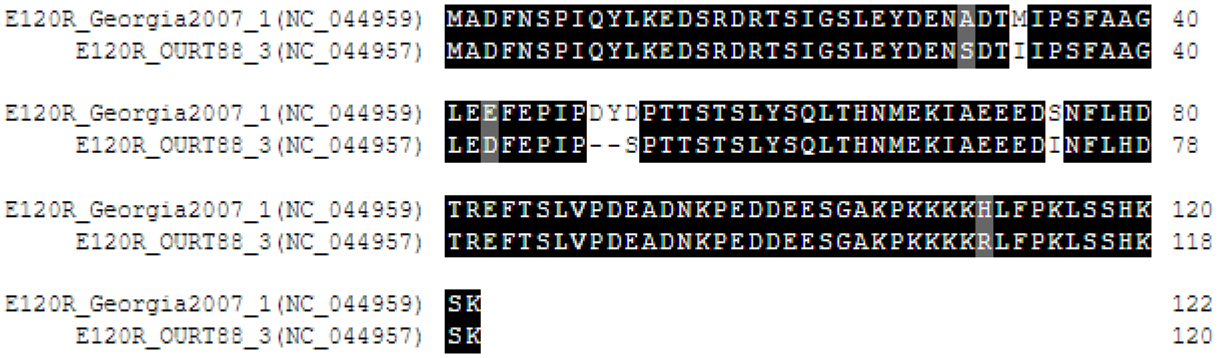

Figure 11. Multiple sequence alignment of E120R/p14.5 amino acid sequences from Georgia2007/1 and OURT88/3. These sequences have 93.4% identity.

```
M1249L_Georgia2007_1(NC_044959)  MEEVITIAQIVHRGTDILSLNNEEIEALVDEIYSTLKGSN  40
     M1249L_OURT88_3(NC_044957)  MEEVITIAQIVHRGTDILSLNNEEIEALVDEIYSTLKGSN  40

M1249L_Georgia2007_1(NC_044959)  DIKNIRLIDFLFTLKDFVNHVRAEQSKLPDLSMPIEAYIR  80
     M1249L_OURT88_3(NC_044957)  DIKNIRLIDFLFTLKDFVNHVRAEQSKLPDLSMPMEAYIR  80

M1249L_Georgia2007_1(NC_044959)  QLLVDPDVVPIVSEKKKELRVRPSTRKEIFLINGTHLAVP  120
     M1249L_OURT88_3(NC_044957)  QLLVDPDVVPIVSEKKKELRVRPSTRKEIFLINGTHLAVP  120

M1249L_Georgia2007_1(NC_044959)  AEAPIEIYGLKLRLKTFSPQCFMRMAEIGSFSPETLGYVA  160
     M1249L_OURT88_3(NC_044957)  AEAPIEIYGLKLRLKSFSPQCFMRMAEIGSFSPETLGYVA  160

M1249L_Georgia2007_1(NC_044959)  SGANLTNFIRVFMKCVDQETWKKNGEGVVVTTKENIIQFT  200
     M1249L_OURT88_3(NC_044957)  SGANLTNFIRVFMKCVDQETWKKNGEGVVVTTKENIIQFT  200

M1249L_Georgia2007_1(NC_044959)  HQYIELYKFLRSGGHSWLINRLAEEMVHRKLDREDQGSHI  240
     M1249L_OURT88_3(NC_044957)  HQYIELYKFLRSGGHSWLINRLAEEMVHRKLDREDQGSHI  240

M1249L_Georgia2007_1(NC_044959)  SNIVETEEIEPEENIKRVIFFLKELSTMYSVSPVFTSGYM  280
     M1249L_OURT88_3(NC_044957)  SNIVETEEIEPEENIKRVIFFLKELSTMYSVSPVFTSGYM  280

M1249L_Georgia2007_1(NC_044959)  PLLYDLYRAGYLEVLWNPVEQKFLQHAEQREKEQMILQQV  320
     M1249L_OURT88_3(NC_044957)  PLLYDLYRAGYLEVLWNPVEQKFLQHAEQREKEQMILQQV  320

M1249L_Georgia2007_1(NC_044959)  DMKLTEVITQARQYFKIMEEKIGRVQSDAIREILTMEGKV  360
     M1249L_OURT88_3(NC_044957)  DMKLTEVITQARQYFKIMEEKIGRVQSDAIREILTMEGKV  360

M1249L_Georgia2007_1(NC_044959)  DDPNSILQEVIKACGKQEAELITTEYLNIKKQWELQEKNA  400
     M1249L_OURT88_3(NC_044957)  DDPNSILQEVIKACGKQEAELITTEYLNIKKQWELQEKNA  400

M1249L_Georgia2007_1(NC_044959)  CAHLKLVKQLRSGLQYAELLKVLESIRVLYKEKNNTTNWN  440
     M1249L_OURT88_3(NC_044957)  CAHLKLVKQLRSGLQYAELLKVLESIRVLYKEKNNTTNWN  440

M1249L_Georgia2007_1(NC_044959)  LCKACGFKLLCPHVDMLIQLQAAEASYDTMRTKLMKFSGI  480
     M1249L_OURT88_3(NC_044957)  LCKACGFKLLCPHVDMLIQLQAAEASYDTMRTKLMKFSGI  480

M1249L_Georgia2007_1(NC_044959)  NKEKENNQGLIYSYFCKICGEELAHFIQEDRTADVGIIGD  520
     M1249L_OURT88_3(NC_044957)  NKEKENNQGLIYSYFCKICGEELAHFIQEDRTADVGVIGD  520

M1249L_Georgia2007_1(NC_044959)  LNSKLRVFIWQETMKACTFIHFGKLVDVKQFANIAVNVCL  560
     M1249L_OURT88_3(NC_044957)  LNSKLRIFIWQETMKACTFIHFGKLVDVKQFANIAVNVCL  560

M1249L_Georgia2007_1(NC_044959)  PLVYSIENIKKEEDYDPLTQLYAVIYIYAYILNLIYSSQK  600
     M1249L_OURT88_3(NC_044957)  PLVYSIENIKKEEDYDPLTQLYAVIYIYAYILNLIYSSQK  600

M1249L_Georgia2007_1(NC_044959)  NKEFLTITIHGMKADSSLNAYVTFLLEKMMQQYSGIINQL  640
     M1249L_OURT88_3(NC_044957)  NKEFLTITIHGMKADSSLNAYVTFLLEKMMQQYSGIINQL  640

M1249L_Georgia2007_1(NC_044959)  SEITDQWIANNFREAFKKIIHQNGLQGLSVQDDTKVLLTE  680
     M1249L_OURT88_3(NC_044957)  SEITDQWIANNFREAFKKIIHQNGLQGLSVQDDTKVLLTE  680

M1249L_Georgia2007_1(NC_044959)  ILLDPMYDYAATVARIDGSIPMHKPRTPKEAEYEFKTVIG  720
     M1249L_OURT88_3(NC_044957)  ILLDPMYDYAATVARIDGSIPMHKPRTPKEAEYEFKTVIG  720
```

Figure 12. Multiple sequence alignment of M1249L amino acid sequences (1-720) from Georgia2007/1 and OURT88/3. These sequences have 99.4% identity.

```
M1249L_Georgia2007_1(NC_044959) RTPAELLSQKEFYDKIYTSKYRPDFTQLTRLNDIYFQEES 760
     M1249L_OURT88_3(NC_044957) RTPAELLSQKEFYDKIYTSKYRPDFTQLARLNDIYFQEES 760

M1249L_Georgia2007_1(NC_044959) LRVWWGGRDEEKTSTLIYLRAYELFLKYLQNAPNFNSELA 800
     M1249L_OURT88_3(NC_044957) LRVWWGGRDEEKTSTLIYLRAYELFLKYLQNAPNFNSELA 800

M1249L_Georgia2007_1(NC_044959) EFKTYENAYGEQKALLAQQGFYNIFDPNTGRADQRTRLFE 840
     M1249L_OURT88_3(NC_044957) EFKTYENAYGEQKALLAQQGFYNIFDPNTGRADQRTRLFE 840

M1249L_Georgia2007_1(NC_044959) YKRLPISTLYDERGLPHKWTIYVYKAVDSSQKPAEIEVTR 880
     M1249L_OURT88_3(NC_044957) YKRLPISTLYDERGLPHKWTIYVYKAVDSSQKPAEIEVTR 880

M1249L_Georgia2007_1(NC_044959) KDVIKKIDNHYALADLRCSVCHVLQHEVGQLNIKKVQTAL 920
     M1249L_OURT88_3(NC_044957) KDVIKKIDNHYALADLRCSVCHVLQHEVGQLNIKKVQTAL 920

M1249L_Georgia2007_1(NC_044959) KASLEFNTFYAFYESRCPKGGLHDFQDKKCVKCGLFTYII 960
     M1249L_OURT88_3(NC_044957) KASLEFNTFYAFYESRCPKGGLHDFQDKKCVKCGLFTYII 960

M1249L_Georgia2007_1(NC_044959) YDHLSQPELVHDYYNNYKDQYDKEKMSIRSIQIKKDMTTP 1000
     M1249L_OURT88_3(NC_044957) YDHLSQPELVHDYYNNYKDQYDKEKMSIRSIQIKKDMTTP 1000

M1249L_Georgia2007_1(NC_044959) STETQPKPPQEPWTFDYGKIIKTAKILDISPAVIEAIGAM 1040
     M1249L_OURT88_3(NC_044957) SSETQPKPPQEPWTFDYGKIIKTAKILDISPAVIEAIGAM 1040

M1249L_Georgia2007_1(NC_044959) EGRSYADIREGQGAPPPPTSMDDPRLMAVDSAVRIFLYNY 1080
     M1249L_OURT88_3(NC_044957) EGRSYADIREGQGAPPPPTSMDDPRLMAVDSAVRIFLYNY 1080

M1249L_Georgia2007_1(NC_044959) NCLRHVSTFNKPPIHVERLVKHLSYEEKEDLEKVLPNVVN 1120
     M1249L_OURT88_3(NC_044957) NCLRHVSTFNKPPMHVERLVKHLSYEEKEDLEKVLPNVVN 1120

M1249L_Georgia2007_1(NC_044959) EYHTTFKHLRVTDPASALLYSIEFLCISFLTLYEIKEPSW 1160
     M1249L_OURT88_3(NC_044957) EYHTTFKHLRVTDPASALLYSIEFLCVSFLTLYEIKEPSW 1160

M1249L_Georgia2007_1(NC_044959) VVNIVREFALTELNTIIQSEKLLSKPGAFNFMIFGEDFVC 1200
     M1249L_OURT88_3(NC_044957) VVNIVREFALTELNTIIQSEKLLSKPGAFNFMIFGEDFVC 1200

M1249L_Georgia2007_1(NC_044959) SGEDSSMDDISAYSSPGLFGEDIIDRLDDPFSIEDVDISL 1240
     M1249L_OURT88_3(NC_044957) SGEDSSMDDISAYSSPGLFGEDIIDRLDDPFSIEDVDISL 1240

M1249L_Georgia2007_1(NC_044959) DVLDNLAPQ                                1249
     M1249L_OURT88_3(NC_044957) DVLDNLAPQ                                1249
```

Figure 13. Multiple sequence alignment of M1249L amino acid sequences (721-1249) from Georgia2007/1 and OURT88/3. These sequences have 99.4% identity.

Supplementary Materials and Methods

Confocal staining and imaging

Vero African green monkey cells were used for confocal imaging as these enable better demarcation of cell morphology and have been used previously in our group (add DOI: 10.1099/jgv.0.001637). Briefly, cells were seeded at a density of 5 x 10^4 cells before transfection with plasmids and TransIT-LT1. After 24 hours incubation post-transfection, cells were fixed in 4% paraformaldehyde for 20 min before three washes with 1xPBS. For cells that were infected with ASFV (BA71V), cells were infected at an multiplicity of infection of 2, before washing and fixing 20 hours after infection. Cells were permeabilized with 0.2% (v/v) Triton-X in 1x PBS, then blocked with block buffer consisting of 10% (v/v) goat sera, 0.2% (v/v) NaN3 and 0.2% (v/v) fish skin gelatin (Sigma-Aldrich, USA) in 1x PBS. Primary antibodies from pig sera were first pre-adsorbed on untransfected cells that were treated in the same manner. Coverslips were incubated for 1h with the following primary antibodies: pre-adsorbed pig sera (1:800) and mouse anti-nanoluciferase (1:200, Promega, USA) or anti-B646L/p72 (1:10, 4H3, in-house hybridoma supernatant) diluted in block buffer. After 3 washes with 1x PBS, coverslips were incubated with the appropriate secondary antibodies: anti-pig IgG Alexa Flour 488 (1:250, Southern Biotech, USA) and/or anti-mouse IgG Alexa Flour 568 (1:200, Invitrogen, USA) diluted in block buffer. Coverslips were washed another three times with 1x PBS before DNA staining with DAPI or TOPRO. Coverslips were mounted with Vectashield and imaging on a Leica SP8 confocal microscope.

Western blot analysis

Protein expression was confirmed with sodium dodecyl sulfate-polyacrylamide gel electrophoresis and western blot analysis. Human embryonic kidney cells (HEK293T) were used as these have a high transfection efficiency that is suitable for transient expression of exogenous proteins. Briefly, cell lysates from plasmid transfected HEK293T cells were prepared for separation on 4-12% NuPAGE Bis-Tris protein gels (Invitrogen, USA) with NuPAGE LDS sample buffer and reducing agent. Proteins were then transferred onto PVDF membranes (Cyvita, USA). Mouse anti-nanoluciferase antibodies (1:400, Promega, USA) were used for protein detection. Membranes were blocked with 5% skim milk (w/v) in 0.5% (v/v) PBS-Tween20 (PBS-T). Primary antibodies were diluted in 5% bovine serum albumin (BSA, w/v) in PBS-T and incubated with the blocked membranes for 1h with agitation. Membranes were washed three times with PBS-T before detection of primary antibodies with goat anti-mouse IgG HRP-conjugated antibodies (1:2000, BioRad, USA) diluted in 5% BSA in PBS-T. Protein visualization was achieved with SuperSignal West Pico PLUS chemiluminescent substrate (Thermo Scientific, USA) on the G:BOX XX6 (Syngene, USA).

Multiple alignments of ASFV protein sequences

Multiple sequences alignments generated from the selected ASFV capsid protein amino acid sequences derived from Georgia 2007/1 (GenBank No. NC_044959) and OURT88/3 (GenBank No. NC_044957) were aligned with EMBOSS Needle (EMBL-EBI). The FASTA output was shaded with the Multiple Align Show (http://www.bioinformatics.org/SMS/multi_align.html).

Table 1. DNA sequences of recombinant Nluc tagged ASFV protein open reading frames. Open reading frames were inserted into the pNeoSec vector containing a Nluc tag with a multiple cloning site containing a *HindIII* (Capitalised) and *AscI* cloning site (underlined). Start codon highlighted in bold.

B646L/p72
AAGCTTgccacc**atg**gtaccagcatctggcggagcctttgtctgatcgccaacgacggaaaggccgacaagatcattctggcccaggacctgctgaacagccgcatcagcaacatcaagaacgtgaacaagagctacggcaagcccgatccagagccaacactgagccagatcgaagagacacacctggtgcacttcaacgcccacttcaagccctacgtgccagtgggcttcgagtacaacaaagtgcgcccacacaccggcacacccacactgggaaacaagctgaccttcggcatccctcagtacggcgacttcttccacgacatggtcggacaccacatcctgggagcctgtcacagctcttggcaggatgccccaatccagggaacctctcagatgggagcacacggccagctgcagacattccctagaaacggctacgactgggacaaccagacaccactggaaggcgccgtgtacacactggtggacccattcggaaggccaatcgtgccaggcaccaaaaacgcctaccgcaacctggtgtactactgcgagtacccaggcgagcgcctgtacgagaacgtgcgcttcgacgtgaacggcaacagcctggacgagtacagcagcgacgtgacaaccctcgtgcgcaagttctgtatccccggcgacaagatgaccggctacaagcacctcgtgggccaagaggtgtcagtggaaggcacatctggacccctgctgtgcaacatccacgacctgcacaaaccccaccagagcaagccaatcctgaccgacgagaacgacacccagagaacctgcagccacactaaccccaagtttctgagccagcacttccccgagaacagccacaacatccagacagccggcaagcaggacatcaccccaatcaccgatgccacctacctggacatcaggcgcaacgtgcactacagctgcaacggcccacagacacccaagtactaccagccacctctggctctgtggatcaagctgcgcttctggttcaacgagaatgtgaacctggctatcccctccgtgtctatccctttcggcgagcgcttcatcaccatcaaactggccagccagaaggacctggtcaacgagttcccaggactgttcgtccgccagagccgctttattgctggacgaccaagccgccgcaacatccgcttcaagccatggtttatcccaggcgtgatcaacgagatcagcctgaccaacaacgagctgtacatcaacaacctgttcgtgaccccagagatccacaacctcttcgtcaagcgcgtgcggttctctctgatccgcgtgcacaaaacccaagtgacccacaccaacaacaaccaccacgatgagaagctgatgagcgccctgaagtggcccatcgagtacatgttcatcggcctgaagccaacctggaacatctccgaccagaatccacaccagcaccgcgactggcacaagttcggacacgtggtcaacgccatcatgcagccaacacaccacgccgaaatcagcttccaggacagggatacagccctgccagacgcctgtagctccatcagcgatatcagccccgtgacataccccatcacactgcccatcatcaaaaacatcagcgtgacagcccacggcatcaacctgatcgacaagttcccatccaagttctgcagcagctacatccccttccactacggcggcaatgccatcaagacaccagatgacccaggcgccatgatgatcaccttcgctctgaagccccgcgaggaataccagccaagcggacacatcaacgtgtcccgcgccagagagttctacatcagctgggacacagactacgtgggcagcatcacaacagccgacctggtcgtgtctgccagcgccatcaattttctgctgctccagaacggcagcgccgtgctgagatattctacacggcgcgcc
M1249L
AAGCTTgccaccatggaggaggtcatcaccatcgcccagatcgtgcacagaggcacggatatcctgagtctgaacaatgaagaaatcgaggccctggtggacgagatttactccacgttaaaagggagcaatgatataaagaatatacggctgatcgacttcctgtttactctgaaagatttgtgaaccatgtcagagcggagcagagcaagctgcctgacctcagcatgcccatagaggcctacatccggcagttattagtagatcctgatgtggttccaattgtctccgagaagaagaaagagttgagggtgcggcctagcacccgaaaggaaatatttcttatcaatggcactcatcttgcggtcccagccgaggcgcccatcgagatttatgggctgaaactccgtctgaagactttcagtccccagtgcttcatgagaatggctgaaattggcagcttttcccctgaaactttaggctatgtggcctctggggccaatctgactaatttcattcgtgtattcatgaagtgtgtggaccaggagacctggaagaaaaatggggaaggggtcgtcgtgaccacaaaggaaaatatcatacagttcacgcaccagtatatcgaactttataagtttctccgatcaggcggccatagctggctcattaaccgacttgcagaggagatggtgcatcgcaagctggatcgagaggaccagggctcgcacatcagcaatattgtggagacggaagaaatcgagcccgaggagaacattaaaagagtgatctttttttaaaagagctatctacaatgtacagtgtttcacccgtgtttacttctggctacatgcctctattgtatgatctttaccgggccggatacctggaggttctgtggaaccccgtcgagcaaaaattcctgcaacatgcagagcagagagaaaaggagcagatgatcctgcaacaggtggacatgaaactgactgaagtcattacgcaggccaggcagtatttaagatcatggaggaaaaaattggccgcgtccagtctgatgccattcgggagatcctcaccatggaaggaaaggtggatgatcccaactccatactgcaggaagtaatcaaggcctgtggcaagcaagaagctgagttgataaccacagaatacctcaatattaagaaacaatgggaactgcaagaaaagaacgcctgtgcccacctaaagttagttaagcaattgcgctcaggcctgcagtatgccgaactccttaaggttctggaatctatcagagtgctctataaggaaaaaaataatacaaccaactggaatctgtgcaaagcctgtggatttaagctactgtgcccgcacgtggacatgctgattcagctgcaggctgccgaagcttcctacgacaccatgaggaccaaactgatgaagttctctggtatcaataaagagaaagaaaacaaccagggactgatatacagttatttttgcaagatttgtggagaggagttagctcatttcatccaggaggaccgcacagcagatgtaggaatcatcggggacctaaacagcaagctgcgggtgttcatctggcaggagaccatgaaggcatgcacattcattcacttcggcaaactcgtagatgtcaagcagtttgccaacattgcggtgaatgtatgcctgccgctggtgtattcaatagagaatataaaaaaagaggaagattatgacccccttactcagctctacgcagtgatctacatttatgcctatattctgaacctgatctacagctctcagaagaacaaggaatttcttactatcacgatacatggcatgaaagccgactcctccctcaacgcttacgtaacattcttgctggagaaaatgatgcagcagtacagcgggattatcaatcaactttcggagattacagatcagtggatagcaaataacttccgggaggccttcaaaaaaatcattcaccaaaacggtcttcaaggactcagtgtacaggacgacacaaaggttctcttaactgagattctgctcgacccgatgtatgattacgctgctaccgtcgccagaatagacggctccatccccatgcacaaacccaggacacctaaagaggccgagtatgagtttaagactgtgattggaaggactcccgctgagttattgtcccagaaagaattttatgataagatctacacgtcgaagtacagaccagatttacccaactgacgaggttaaatgacatctattttcaggaagaaagcctgcgggtgtggtggggtggaagggatgaggagaaaacatcaactctgatctacttgcgggcatatgagctctttctgaaatacctccagaacgccccaaatttcaacagtgaattggctgagttcaaaacatatgagaacgcttacggcgaacaaaaggccctgttggctcagcaggggtttctacaacatctttgacccgaatactgggagggcagaccagcgcactcgcctctttgaatacaaaaggctacccatctccacccctttatgatgaacggggactgccacacaagtggaccatctatgtttacaaagcagttgacagttctcagaagccagcagaaatagaggtcaccagaaaggatgttattaaaaagatagacaaccactacgcgctggctgaccttagatgctcggtgtgtcacgtcttacagcacgaggtgggtcagctgaatatcaaaaaagtgcagaccgctttgaaggctagtctggagttcaacacattctatgcgttctatgagtcacgctgccccaaaggaggacttcatgacttccaggacaagaagtgtgtcaaatgcggtctcttcacttacatcatatatgaccacctgagccagcctgagctggtacacgactactacaacaactacaaggaccagtacgataaggagaagatgagcattcgaagcatacaaattaagaaggatatgacgacgccaagtaccgaaacacagcccaagcctcctcaggaaccctggacatttgactacgggaaaatcattaagaccgccaagattttggatatctccccagccgtcattgaggcaatcggagccatggaagggcgttcctatgcagacatccgcgaggggcagggtgcaccgccaccaccgaccagtatggacgatccacgcctcatggccgttgacagcgcagtaagaattttcctttataattacaactgtctccgtcatgtctcgaccttcaataaacccccctattcatgtggaaaggttggtcaaacatctctcatatgaagagaaagaggacctggagaaagtcttgcctaacgtggtgaatgagtaccacaccacattcaagcacttgagggttacagaccctgccagcgcactactctactccatcgagtttctctgcatcagctttctaacactgtacgaaatcaaggagccatcatgggtggtgaacattgtgagggagtttgcgctcacagagctgaataccatcatccaatctgaaaagctcctgtccaaacccggggcttttaactttatgatttcggtgaagatttcgtgtgttctggcgaagattcttccatggatgatatatcagcttactcttccccgggcctttcggcgaggacattattgatagactggacgaccccttctccattgaagatgtggacatcagtttagatgtgttggataacttggcccctcaagggcgcgcc
D117L
AAGCTTgccaccatggacacagaaacttcacctttgctcagtcacaacctctctacaagggaaggaatcaagcagtctactcaaggtcttctagctcataccattgccaagtacccagggaccacagccatccttctgggcatcctcatcctgttgatcatcattttaataattgtggctattgtttactacaaccgcacgattgactgcaaatcctccatacccaagcctccacccagctattatgtgcagcagccagagccccaccatcatttcctgtcttcttccggaaaagaaaaaattcaacctccctgcagagccacatcccgagtgatgagcagctggcagagctggcgcacagcgggcgcgcc

B438L
AAGCTTgccacc**atg**taccatgactatgcaagcaagctcctcgcggattacaggagtgatccaccactgtgggagtcagatttacccagacataatcgctacagcgacaacatcctgaac<br>agcaggtactgtggaaataaaaatggcgctgcgcctgtctacaatgaatataccaatagccctgagaaggctgaaaaagggctgcagctgtctgacttgcgtaacttcagtttcatgctcaac<br>ccacaacacaagaatattggatatggggacgcccaggatttagagccgtattctagtatccctaagaacaaactcttcaatcacttcaaaaaccatcggccggccttctccactcatactgaaaa<br>tttgatccggcggaatgttgtaagaaccgagaagaagaccttccctcaggtggccagtctgaagggcacgcagaagaactgcctgactcagccctcttcgcttccttccctaaaaaccccaa<br>gaactcctcagttccgtcaacacgcttcagcgagcacaccaaatttttttcttacgaggaccttccaaagttaagaacgaaaggaacaataaaacatgagcagcacttgggtgaccagatgcc<br>aggccagcattataacggctatatcccgcacaaagatgtctacaatatcctctgtcttgcacacaacctgcccgcatcagtggaaaaagggattgctgggcgaggaataccactgggcaacc<br>ctcacgtgaagccaaatattgaacaagagctgataaagagcacatcaacatatacagatgtccccatgctaggccccctccctcccaaagacagccaacacggccgggagtaccaggaattt<br>tccgctaatagacacatgttgcaggtgagcaatatcctgcactccgtgtttgcaaatcattccatcaagccgcagattctggaggatattccagtgcttaacgctcagctcacgtcgataaaacct<br>gtaagccccttcctgaacaaggcctatcaaacgcactacatggagaacattgtcaccctggtgcccaggtttaaatccattgcgaactattcttcccccatccccaactactccaagaggaacag<br>tggacaggccgaatactttgatacttcaaagcagaccatcagtcgacataacaattatatacctaagtacaccgggggcattggtgattctaagctggacagcaccttccctaaagactttaatg<br>cctcatctgttcccctgacttccgccgaaaaggaccacagcctccgcggtgacaattctgcctgctgcatcagcagtatctccccatctttggggcgcgcc

E120R
AAGCTTgccacc**atg**gccgacttcaacagccccatccagtacctgaaagaagactcgagagacaggacatctatagggagtttggaatatgacgagaatgctgataccatgatcccttc<br>ctttgctgcaggactggaggaatttgagcccattccagattatgatcctacgacttcaacatccctctacagccagctgactcacaacatggagaagattgccgaggaggaggacagcaactt<br>cctgcatgacacccgggagttcaccagcttagtgcctgatgaggcggacaataaaccagaagatgatgaagaaagtggcgccaagcccaagaaaaagaagcatctctttccgaaactttca<br>tctcacaagtccaaagggcgcgcc

B602L
AAGCTTgccacc**atg**ggtaccgccgagttcaacatcgacgagctgctgaagaacgtgctggaagatcccagcaccgagatcagcgaggaaaccctgaagcagctgtaccagcgcac<br>aaacccctacaagcagttcaagaacgacagccgcgtggccttctgcagcttcaccaatctgcgcgagcagtacatccgccgcctgatcatgaccagcttcatcggctacgtgttcaaggccct<br>gcaagagtggatgcccagctacagcaagcccacacacaccaccaagacactgctgagcgagctgatcaccctggtggacaccctgaaacaagagacaaacgacgtgcccagcgagagc<br>gtggtcaacaccatcctgtctatcgccgacagctgcaagacccagacacagaagtccaaagaggccaagacaacaatcgacagcttcctgcgcgaacacttcgtgttcgacccaaacctgca<br>cgcccagagcgcctacacatgtgccgacacaaacgtggacacctgtgccagcatgtgcgccgacaccaatgtggatacttgcgcctctatgtgtgccgatacgaatgtcgacacatgcgcca<br>gcacctgtacctccaccgagtacacagatctggccgatccagagcgcatcccactgcacatcatgcagaaaacactgaacgtgccaaacgagctgcaggccgacatcgacgccatcacaca<br>gacaccacagggatatagagccgccgctcacatcctccagaacatcgagctgcaccagagcatcaagcacatgctcgaaaaccccagggccttcaagcccatcctgttcaacaccaagatca<br>cccgctacctgagccagcacatcccaccacaggacaccttctacaagtggaactactacatcgaggacaactacgaggaactgagggccgccaccgagagcatctacccagagaaacccg<br>acctggaattcgccttcatcatctacgacgtggtggacagcagcaaccagcagaaggtggacgagttctactacaagtacaaggaccagatcttcagcgaggtgtccagcatccagctcgg<br>caattggactctgctgggcagcttcaaagccaaccgcgagcgctacaactacttcaaccagaacaacgagatcatcaaacgcatcctggaccgccacgaagaggacctgaagatcggcaa<br>agagatcctgaggaacaccatctaccacaagaaggccaagaacatccaagagacaggcccagacgctcccggcctgtccatctacaacagcaccttccacaccgacagcggcatcaaggg<br>actgctgtccttcaaagagctgaaaaacctggaaaaggccagcggcaacatcaagaaagcccgcgagtacgacttcatcgacgattgcgaggaaaagatcaagcagctcctgagcaaag<br>agaatctgaccccagacgaggaaagcgaactgatcaagaccaagaagcagctcgacaacgccctggaaatgctgaatgtgcccgacgacacaatcagagtggatatgtgggtcaacaac<br>aacaacaagctggaaaaagagatcctctacaccaaggccgagctggggcgcgcc

Table 2. DNA sequence of recombinant Nluc with a 5′ multiple cloning site (MCS) containing a *HindIII* (Capitalised) and *AscI* cloning site (underlined). Start codon highlighted in bold.

MCS-Nluc
AAGCTTgccacc**atg**gtaccaggttctcggcgcgccgtgttcacactcgaagatttcgttggggactggcgacagacagccggctacaacctggaccaagtccttgaacagggaggtg<br>tgtccagtttgtttcagaatctcggggtgtccgtaactccgatccaaaggattgtcctgagcggtgaaaatgggctgaagatcgacatccatgtcatcatcccgtatgaaggtctgagcggcga<br>ccaaatgggccagatcgaaaaaatttttaaggtggtgtaccctgtggatgatcatcactttaaggtgatcctgcactatggcacactggtaatcgacggggttacgccgaacatgatcgactat<br>ttcggacggccgtatgaaggcatcgccgtgttcgacggcaaaaagatcactgtaacagggaccctgtggaacggcaacaaaattatcgacgagcgcctgatcaaccccgacggctccctgc<br>tgttccgagtaaccatcaacggagtgaccggctggcggctgtgcgaacgcattctggcgcgcgccaagcaccaccatcaccatcactaa

References

1. Goatley, L. C.; Nash, R. H.; Andrews, C.; Hargreaves, Z.; Tng, P.; Reis, A. L.; Graham, S. P.; Netherton, C. L., Cellular and Humoral Immune Responses after Immunisation with Low Virulent African Swine Fever Virus in the Large White Inbred Babraham Line and Outbred Domestic Pigs. *Viruses* **2022,** 14, (7).
